# SUMOylation pathway alteration coupled with downregulation of SUMO E2 enzyme at mucosal epithelium modulates inflammation in inflammatory bowel disease

**DOI:** 10.1098/rsob.170024

**Published:** 2017-06-28

**Authors:** Salman Ahmad Mustfa, Mukesh Singh, Aamir Suhail, Gayatree Mohapatra, Smriti Verma, Debangana Chakravorty, Sarika Rana, Ritika Rampal, Atika Dhar, Sudipto Saha, Vineet Ahuja, C. V. Srikanth

**Affiliations:** 1 Laboratory of gut inflammation and infection biology (LGIIB), Regional Centre for Biotechnology, 3rd milestone Gurgaon Faridabad Expressway, Faridabad, India; 2 Department of Gastroenterology, Manipal University, Manipal, Karnataka, India; 3 Kalinga Institute of Industrial Technology, Bhubaneswar, Odisha, India; 4 All India Institute of Medical Sciences, Ansari Nagar East, New Delhi, India; 5 Functional interactomics laboratory, Bose Institute Kolkata, P 1/12, C.I.T Road, Scheme VII M, Kolkata 700054, India; 6 National Institute of Immunology, New Delhi, India; 7 Mucosal Immunology and Biology Research Center, Massachusetts General Hospital, Charlestown, Boston, MA, USA

**Keywords:** colitis, inflammation, post-translational modification, SUMOylation, epithelial signalling

## Abstract

Post-translational modification pathways such as SUMOylation are integral to all cellular processes and tissue homeostasis. We investigated the possible involvement of SUMOylation in the epithelial signalling in Crohn's disease (CD) and ulcerative colitis (UC), the two major forms of inflammatory bowel disease (IBD). Initially in a murine model of IBD, induced by dextran–sulfate–sodium (DSS mice), we observed inflammation accompanied by a lowering of global SUMOylation of colonic epithelium. The observed SUMOylation alteration was due to a decrease in the sole SUMO E2 enzyme (Ubc9). Mass-spectrometric analysis revealed the existence of a distinct SUMOylome (SUMO-conjugated proteome) in DSS mice with alteration of key cellular regulators, including master kinase Akt1. Knocking-down of Ubc9 in epithelial cells resulted in dramatic activation of inflammatory gene expression, a phenomenon that acted via reduction in Akt1 and its SUMOylated form. Importantly, a strong decrease in Ubc9 and Akt1 was also seen in endoscopic biopsy samples (*N* = 66) of human CD and UC patients. Furthermore, patients with maximum disease indices were always accompanied by severely lowered Ubc9 or SUMOylated-Akt1. Mucosal tissues with severely compromised Ubc9 function displayed higher levels of pro-inflammatory cytokines and compromised wound-healing markers. Thus, our results reveal an important and previously undescribed role for the SUMOylation pathway involving Ubc9 and Akt1 in modulation of epithelial inflammatory signalling in IBD.

## 1. Introduction

Inflammatory bowel disease (IBD), which includes ulcerative colitis (UC) and Crohn's disease (CD), is a chronic inflammatory disorder of the intestine characterized by phases of remission and relapse. UC affects primarily the colon in a contiguous fashion and is characterized by superficial inflammation, whereas CD can affect any part of the intestine and is characterized by transmural inflammation. Genetic predisposition, dysbiosis, environmental factors and aberrant immune responses are key factors in the pathogenesis of IBD. Genome-wide association studies performed in IBD patients have identified 163 genes associated with the pathogenesis of IBD with about a quarter of them being shared between CD and UC [1,2].

Mucosal surfaces involve constant crosstalk between the epithelium, commensal microflora and the local immune cells. Owing to its complexity, even the slightest perturbations can potentially tilt the fine balance, and may result in inflammation and disease. The epithelial cells that are not just a physical barrier contribute a lot in the multi-directional interaction. They regulate the colonization and penetration of luminal microbe in many different ways [3]. The epithelium is also engaged in transducing the information about microbial invasion to underlying lamina propria by secreting a variety of cytokines [4]. Excessive activation of innate and adaptive immune components leads to inflammatory response and tissue destruction in IBD. CD is characterized by a Th1/Th17 phenotype, while UC has been recently recognized as Th2/Th17 disease. At the molecular level, a balance between the anti-inflammatory and pro-inflammatory signals is crucial. During disease, the molecular events that lead to a tilt in the balance are not fully understood. Post-translational modifications (PTMs) can rapidly and reversibly alter functional relevance of the proteome without the requirement of de novo synthesis of large sets of proteins. Understandably, PTM mechanisms including phosphorylation, acetylation and ubiquitination have been known to play a crucial role in IBD [5]. PTM by small ubiquitin-like modifier (SUMO) and its role in IBD has not been investigated. Our study focuses on understanding the importance of SUMO conjugation (or SUMOylation) during IBD, specifically in the context of epithelium.

SUMOylation is highly conserved and is central to the regulation of various cellular processes. Three SUMO paralogues (SUMO1, SUMO2 and SUMO3) are present in mammals [6]. The addition of SUMO to the target substrate requires sequential enzymatic action of E1 enzyme (SAE1/SAE2 heterodimer), E2 enzyme (UbC9) and one of several E3 ligases that act on specific targets. SUMOylation has been shown to participate in several fundamental cellular processes, including replication, transcription and genome maintenance [7]. Gut pathogens have been shown to target the host SUMOylation machinery and alter the SUMOylation level of host protein(s) [8,9]. Several regulators of the inflammatory cascade have been previously demonstrated to be dependent on SUMOylation for their proper function [7–9]. The important molecule among these involves RelA, a component of the master regulator nuclear factor κB (NFκB) and its repressor, IκBα [10]. In this work, using multiple systems ranging from cell culture model to clinical patient samples, we demonstrate that SUMOylation status of epithelial cells critically modulates the activities of master regulators including the serine–threonine kinase Akt1, which in turn regulate the severity of IBD.

## 2. Material and methods

All chemical unless otherwise specified were obtained from Sigma-Aldrich, USA.

### 2.1. Animal strains

Female C57BL/6 mice aged six to eight weeks were used for the study and kept under controlled temperature. Experiments were carried out in the Small Animal Facility of the NII (National Institute of Immunology). The NII Institutional Animal Ethics Committee approved the study (approval no. IAEC#331/14).

### 2.2. Induction of colitis

Experimental colitis was induced by adding dextran–sulfate–sodium (DSS; 40 kDa, Sigma, USA) to the autoclaved drinking water at 2.5% concentration (w/v). Animals were monitored daily for their stress and rectal bleeding.

### 2.3. Haematoxylin and eosin staining and immunohistochemistry

Proximal colon sections were fixed in 10% formalin buffer overnight and embedded in paraffin. Five micrometre thick sections were cut onto glass slides and processed for haematoxylin (Sigma) and eosin (Sigma) staining. The slides were dried and mounted using DPX mountant (Sigma) and images were taken using a Nikon (NY, USA) inverted fluorescence microscope. For immunohistochemistry (IHC), tissue sections were washed using 1× PBS three times (5 min each). Further, the endogenous peroxidase activity was quenched by treating the tissue with 3% H_2_O_2_ for 20 min. The tissue sample was again washed using 1× PBS twice (5 min each), then blocked with 5% goat serum at room temperature for 1 h. The sections were incubated with anti-Ubc9 (1 : 200; Abcam, USA) prepared in 5% goat serum overnight in a moist chamber. Sections were washed with 1× PBS five times for 5 min each and incubated with HRP-conjugated secondary antibody (1 : 200) (Invitrogen, USA) for 2 h prepared in 5% goat serum at room temperature in a moist chamber. The tissues were washed three times using 1× PBS, DAB substrate (Sigma, USA) was added and the reaction was stopped by keeping the slides under running tap water. The slides were counterstained using haematoxylin for 10 s and then washed with running milliQ water. The slides were visualized on a compound microscope.

### 2.4. Mouse primary epithelial cells

Proximal and caecal parts of the colon were isolated and cut longitudinally using sterile scissors. The opened colon was washed with ice-cold PBS containing the antibiotics gentamicin 50 µg ml^−1^ (Sigma) and penicillin–streptomycin 100 U ml^−1^ (Gibco, USA) for 10 min inside the laminar hood. Mucus from the surface was removed gently using a cell scraper with soft rubber. After the mucus removal, cells were treated with 30 mM EDTA for 30 min. Cells were washed three times in DPBS solution and seeded on six-well culture plates coated with collagen. Cells were grown in Dulbecco modified Eagle medium (DMEM) media with added 2.5% fetal bovine serum (FBS), 10% insulin (Sigma) for 72 h in a BOD incubator. Cells were treated with AKT1 inhibitor (Sigma), *S*-adenosyl methionine (SAM) (Biovision, USA) and listeriolysin (LLO) (Sigma). For primary mouse epithelial cells, SAM 2 µM (24 h treatment) and LLO 6 nM (1 h treatment) were used.

### 2.5. Sample size estimation

Our fundamental step was to design a large value of computational power (at least 80%) with effective sample size within the available resources and ethical considerations. The variance or standard deviation for sample size calculation was obtained from our pilot study. For every differential expression analysis, the *p*-value was calculated. To this effect we required (*n* = 22, power = 80% and *α* = 5% per group), using Systat software for two mean calculations. Our ultimate goal was to establish the role of the SUMO pathway by examining a large sample size to gain understanding of the SUMOylation level in IBD.

### 2.6. Human colonic biopsy samples

A total of 66 patients in the UC, CD and control groups were included in the study, from the All India Institute of Medical Sciences (AIIMS). Samples were collected from 22 patients in the UC, CD and control (IBD suspected) groups with age over 18 and below 60 years. Inclusion criteria for UC were: (i) patients with suggestive history and characteristic endoscopic and histological findings of UC (ECCO consensus statement) as enrolled from the Inflammatory Bowel Disease Clinic at the Department of Gastroenterology, AIIMS; (ii) patients with an ulcerative colitis disease activity index (UCDAI) of mild to moderate; and (iii) patients with pan colitis or left-sided colitis. Exclusion criteria for UC were: (i) patients with proctitis; (ii) patients already initiated on steroid therapy; and (iii) patients with coexistent disease such as HIV infection, tuberculosis or chronic renal failure. Inclusion criteria for CD were: (i) the diagnosis of CD established on the basis of the presence of characteristic clinical manifestations (chronic diarrhoea, haematochezia, abdominal pain and intestinal obstructive manifestations), endoscopic features (skip lesions, asymmetrical involvement, longitudinal ulcers, aphthous ulcers) and histological evidence (acute or chronic colitis, the presence of inflammation extending beyond muscularis mucosae, lymphoid follicles and granuloma) (ECCO guidelines for diagnosis); and (ii) adult patients with ileocolonic disease and Crohn's disease activity index (CDAI) greater than 150 and less than 350. Exclusion criteria for CD were: (i) patients already initiated on steroid therapy; and (ii) patients with coexistent disease such as HIV infection, tuberculosis or chronic renal failure. Adult patients suspected of having IBD bur showing normal mucosa on colonoscopy were treated as controls. Four to six biopsies from inflamed areas and non-inflamed areas of the colon were also taken from a few patients. Informed consent was obtained from all the patients and the study protocol was submitted to the ethics committee of the institutes: RCB and AIIMS (approval no. IEC/NP/56/2014, OP-16/01.08.2015).

### 2.7. Cell culture

HCT-8 intestinal epithelial cells (ATCC, Manassas, VA, USA) (passages 2–25) were cultured in RPMI medium supplemented with 14 mM NaHCO_3_, 15 mM HEPES buffer (pH 7.4), 2 mM glutamine, 1 mM sodium pyruvate, 40 mg l^−1^ penicillin, 8 mg l^−1^ ampicillin, 90 mg l^−1^ streptomycin and 10% FBS. HeLa cells were cultured in DMEM containing 14 mM NaHCO_3_, 15 mM HEPES buffer (pH 7.5), 8 mg l^−1^ ampicillin, 100 U ml^−1^ penicillin–streptomycin and 10% FBS. Cells were treated with different pharmacological inhibitors: MG-132, 20 μM for 1 h (Sigma), and AKT1 kinase inhibitor, 100 nM for 1 h (Sigma). Recombinant IL-6 (Biovision, USA) was used at 100 ng ml^−1^ for 6 h in different conditions.

### 2.8. Cell transfection

HCT-8 and HeLa cells were used for transfection. One day before transfection, 2.5 × 10^5^ cells were plated in 24-well plates to obtain 50–80% confluence and transfected with Lipofectamine 2000 (Invitrogen) or DharmaFECT (Dharmacon, USA) as per the manufacturer's instructions. Briefly, 1 µg of plasmid or 20 pmol of small interfering RNA (siRNA) (Dharmacon) was diluted in Opti-MEM (Invitrogen). Separately, Lipofectamine 2000 for plasmid and DharmaFECT for siRNA transfection were also diluted and incubated at room temperature for 5 min. Following incubation, the two mixtures were combined and incubated at room temperature for 20 min. This cocktail was added to cells with Opti-MEM and incubated without selection for 24 h.

### 2.9. Western blot

Tissues were lysed in Laemmli buffer (20 mM Tris–HCl, pH 8.0, 150 mM KCl, 10% glycerol, 5 mM MgCl_2_ and 0.1% NP40) supplemented with Halt complete proteases inhibitor. Protein lysates were separated on sodium dodecyl sulfate–polyacrylamide gel electrophoresis (SDS–PAGE) and transferred to nitrocellulose membrane. Blots were probed with antibodies against Ubc9 (Sigma), SUMO1 (Sigma), SUMO2/3 (Sigma), AKT (CST, USA), p-AKT (CST, USA), Akt1 (CST, USA), p-Akt1 (CST, USA), GSK3β (Abcam, USA) and p-GSK3β (CST, USA). Akt1 kinase activity was assessed using a kit (CST, USA).

### 2.10. Quantitative real-time polymerase chain reaction

Total RNA from human, mouse and animal cells was isolated using the Nucleo Spin RNA-II Kit (MN, Germany) according to the manufacturer's protocol. One microgram of each total RNA sample was used to synthesize c-DNA using the i-Script cDNA Synthesis Kit (Bio-Rad, USA). Real-time PCR (qRT-PCR) was performed using a 20 μl reaction volume in a 96-well plate by using i-Taq Syber Green (Bio-Rad) according to the manufacturer's instruction in the Bio-Rad CFX 96™ Real Time Detection System. All reactions were normalized to the housekeeping genes GAPDH and HPRT for human and actin and B2M for mouse samples. See the electronic supplementary material for primer sequences.

### 2.11. Nuclear factor κB polymerase chain reaction array

Total RNA was isolated from HCT-8 cells and mouse colonic tissue using the Nucleo Spin RNA-II Kit (MN, Germany) according to the manufacturer's protocol. One microgram of each total RNA sample was used to synthesize c-DNA using the RT^2^ First Strand Kit (QIAGEN, SA Biosciences, USA) according to the manufacturer's protocol. qRT-PCR array was performed using RT^2^ Syber Green Master Mix (QIAGEN, SA Biosciences, USA) on 96-well plates of Human NFκB Signalling Pathway Array (Cat. No. PAHS-025Z) (QIAGEN, SA Biosciences, USA) and Mouse NFκB Signalling Pathway Array (Cat. No. PAHS-025Y). The PCR reaction was performed following the manufacturer's protocol. The experiment was performed on the Bio-Rad CFX 96™ Real Time Detection System (Bio-Rad). The data were analysed using the RT² Profiler™ PCR Array Data Analysis web-based tool (QIAGEN, SA Biosciences, USA).

### 2.12. Immunoprecipitation

Mouse and human tissue were lysed in immunoprecipitation (IP) lysis buffer (Thermo, USA). Halt proteases complete inhibitor mix (1×) and 20 mM NEM were added to the mixture. Cell debris was removed by centrifugation at 13 000 r.p.m. for 10 min at 4°C. The lysates obtained were incubated with protein G agarose (Thermo) beads for 1 h at 4°C on an end-to-end rotor, followed by centrifugation to remove the beads and the non-specifically bound proteins. The precleared lysate was then used for immune precipitation with their respective antibodies overnight at 4°C on an end-to-end rotor. As isotype controls, IgGs were used. The antibody-bound proteins were then captured using protein G agarose beads and washed five times with IP lysis buffer followed by heating at 95°C for 10 min in laemmli's buffer. For IP, two mice colonic tissue lysates and five patient biopsies were pooled in each category, respectively. Five hundred micrograms protein were used for IP. Isotype IgG (negative control) was also prepared for control samples. Immunoblots were done in triplicate using different mice total colonic lysates.

### 2.13. Enzyme-linked immunosorbent assay

Biopsies were pooled from five patients of each category. The homogenized sample was incubated for 5 min at room temperature to permit the complete dissociation of nucleoprotein complexes. Thereafter, the homogenate sample was centrifuged to remove cell debris and the supernatant was transferred to a new tube. The supernatant was assayed by enzyme-linked immunosorbent assay (ELISA) for IL-6, IFN-γ, TNF-α, TGF-β, IL-10, IL-8 and thymic stromal lymphopoietin (TSLP) using the manufacturer's protocol (R&D, USA; DY206, DY285, DY210, DY240, DY217B, DY1398, respectively).

### 2.14. Liquid chromatography–mass spectrometry/mass spectrometry

LC-MS/MS analyses were performed using colonic epithelial cell lysate pooled from two mice, which was immunoprecipitated with SUMO1 (Santa Cruz, USA) antibody. Five hundred micrograms of protein lysates were used for the analysis. In-gel digestion was carried out using Trypsin Gold (Promega, USA) at 1 : 50 dilution. The samples were purified using C18 SepPak columns (Thermo, USA). The peptide samples were dissolved in 98% milliQ-H_2_O, 2% acetonitrile and 0.1% formic acid. Tandem MS analysis was performed using a 5600 TripleTOF analyzer (ABSCIEX) in Information Dependent mode. Precursor ions were selected across the mass range of 300–1600 *m*/*z*. Protein identification was performed with MASCOT Server 2.3.0 (Matrix Science, UK). User-defined search parameters were as follows: (i) type of search: MS/MS ion search; (ii) enzyme: trypsin; (iii) mass values: monoisotopic; (iv) protein mass: unrestricted; (v) peptide mass tolerance: ±100 ppm; (vi) fragment mass tolerance: ±0.5 Da; (vii) max missed cleavages: 2; (viii) instrument type: ESI_QUAD-TOF; and (ix) taxonomy: *Mus musculus* (mouse). A *MASCOT* search against a concatenated decoy target database from Swiss-Prot consisting of both forward and reverse versions of murine peptides was performed to evolve the cut-off score threshold (*p* < 0.3–0.05). Proteins were selected for further analyses based on peptide score (greater than 16) and were divided into two groups in the all the three samples. Proteins identified by the MASCOT search were used for the analysis with a minimum of one unique peptide.

### 2.15. Scaffold software

Scaffold (version 4.4.8; Proteome Software Inc., Portland, OR, USA) was used to validate MS/MS-based peptide and protein identifications. All MS/MS samples were analysed using MASCOT (Matrix Science, London, UK; v. 2.3.02) and Tandem (The GPM, version CYCLONE (2010.12.01.1). MASCOT and Tandem were set to search the SwissProt_57.15 database (selected for Mus., 16 281 entries) with digestion enzyme trypsin. Fragment ion mass tolerance of 0.50 Da and a parent ion tolerance of 100 ppm were used. Glu->pyro-Glu of the N-terminus, ammonia-loss of the N-terminus, Gln->pyro-Glu of the N-terminus and oxidation of histidine, methionine and tryptophan were specified in MASCOT and Tandem as variable modifications. Peptide identifications were accepted if they could be established at greater than 50.0% probability. The Peptide Prophet algorithm [11] assigned peptide probabilities with Scaffold delta-mass correction. The Scaffold local FDR algorithm assigned peptide probabilities from Tandem. Proteins that contained similar peptides and could not be differentiated based on MS/MS analysis alone were grouped to satisfy the principles of parsimony. Proteins sharing significant peptide evidence were grouped into clusters. The volcano plot was generated after performing the *t*-test using the software between DSS7 and control categories. A Venn diagram was generated from the total number of proteins identified using Scaffold Quantify function for all three categories of mice. Fold changes and *p*-values were also calculated using Scaffold. These data were used for pathway analysis.

### 2.16. Ingenuity pathway analysis

Data were arranged according to *p*-values. The cut-off criteria less than or equal to 0.05 for qRT-PCR and less than or equal to 0.5 for protein data were used. A fold change of greater than 2 and less than 0.5 was considered for qRT-PCR data and greater than 1.5 and less than 0.5 for protein data (upregulated and downregulated) were uploaded in the Ingenuity Pathway Analysis (IPA) (Ingenuity, CA, USA) tool. The canonical pathways were displayed according to percentage overlap with molecules of interest and *p*-values.

### 2.17. Wound-healing assay

Transfected HCT-8 cells were treated with mitomycin C (10 µg ml^−1^) for 6 h. Cell migration was measured using a wound-healing assay [12].

### 2.18. Statistics

All results are expressed as the mean standard error from an individual experiment done in triplicate. Data were analysed with standard two-tailed Student's *t*-test and the Mann–Whitney *U*-test where applicable, with *p*-values of 0.05–0.001 considered statistically significant. We evaluated the statistics with Systat and GraphPad Prism. Correlation studies were analysed with Spearman's correlation.

## 3. Results

### 3.1. SUMOylation pathway is down-modulated in chemically induced colitis in mice

**Figure 1.**
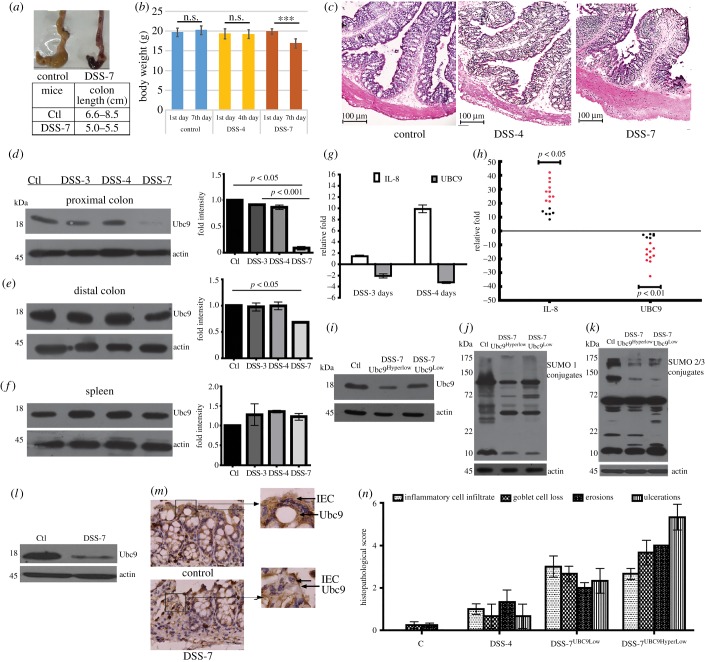
Downmodulation of Ubc9 expression in DSS-colitis mice. (*a*) Top, gross morphology of the caecum and colon of control and DSS-treated mice; bottom, table showing reduced lengths of the colon of control DSS-treated mice (DSS-7) (*n* = 6) compared with the control group. (*b*) Body weight of animals during the course of DSS treatment. (*c*) Haematoxylin and eosin staining of caecum of mice (scale bar, 100 µm) showing exacerbated inflammation in animals. Immunoblots of Ubc9 of lysates from the (*d*) proximal colon, (*e*) distal colon and (*f*) spleen. Densitometry analysis of Ubc9 protein shows lowered Ubc9 in inflamed tissue as represented by means ± s.e.m. from three independent experiments are shown on the right-hand panel in each (*d*,*e*,*f*). Actin was blotted as a loading control. (*g*) qPCR expression of DSS (3 and 4 days, *n* = 5) mice for Ubc9 and IL-8 genes of proximal colon. qPCR data were calculated as average fold change (relative to the control group). B2M and β-actin were used for normalization. (*h*) qPCR data of IL-8 and Ubc9 of DSS-7 mice compared with the control group represented as fold change (relative to control), each black dot represents one mice. Immunoblot representing the downmodulation of Ubc9 (*i*) and global SUMOylation: SUMO-1 proteome (*j*) and SUMO-2/3 proteome (*k*) in DSS-7^HyperLow^ and DSS-7^Low^ compared with control. (*l*) Immunoblot of Ubc9 from epithelial lysates indicating lowered expression. (*m*) Upper panel: IHC of colonic sections of control and DSS-7 mice (scale bar, 100 µm). Magnified regions shown in the inset. (*n*) The histopathology of the colon was quantified by using a disease severity index scale from (0 to 6), with 0 scored normal and a score of 6 showing the most substantial level of disease pathology. The sections were scored blind by a trained pathologist. Statistical significance was assessed (*p* < 0.001–0.05). β-Actin was used as loading control for all immunoblots.

We used the dextran–sodium–sulfate (DSS) mouse model to test the possibility of SUMOylation playing a role in intestinal inflammation. Groups of mice (six animals each) were fed plain water (control mice) or DSS mixed in their drinking water for either 3, 4 or 7 days (hereafter referred to as DSS-3 mice, DSS-4 mice and DSS-7 mice, respectively). Following the treatment, the animals were euthanized and the colonic tissues were isolated for analysis. Among these mice, DSS-7 mice as expected showed severe signs of inflammation (figure 1*a*) and significant weight loss (figure 1*b*). Haematoxylin and eosin staining of intestinal cross-sections of proximal colon of DSS-7 mice showed more neutrophil infiltrates, loss of crypt, colonic wall thickening, epithelial erosion and oedema (figure 1*c*). In contrast, DSS-4 animals did not display any weight loss or discernible signs of inflammation (figure 1*b,c*). SUMO E2 enzyme Ubc9 protein levels of DSS-4 mice in the proximal colon, distal colon and spleen (figure 1*d–f*) were comparable to the control animals. In the proximal colon of DSS-7 animals, a significant downregulation of Ubc9 (*p* < 0.05) (figure 1*d*) was observed relative to control. A mild downregulation of Ubc9 was also seen in the case of the distal colon, while in the spleen such lowering was not observed (figure 1*e*,*f*).

mRNA expression of Ubc9, as seen by qPCR analysis, reflected downregulation on day 4 (−4-fold) and day 3 (−2-fold) relative to control mice (figure 1*g*). These data indicate that Ubc9 downregulation is initiated even before the onset of markers of inflammation. Compared with control mice, the inflammatory cytokine IL-8/CXCL1 mRNA expression was pronounced in DSS-7 mice (*p* < 0.05), while that of Ubc9 was drastically decreased (*p* < 0.01), ranging from 1- to 30-fold downregulation (figure 1*h*). For the sake of convenience of understanding, DSS-7 mice were subgrouped in the following way for further analysis. DSS-7 mice with low Ubc9 (onefold to fivefold) were called DSS-7-Ubc9^Low^ and those with severely downregulated Ubc9 (sixfold and above) were called DSS-7-Ubc9^HyperLow^. The subgrouping was done in this way because the decrease in Ubc9 among the animals was over a wide range. Among DSS-treated mice, Ubc9^HyperLow^ mice outnumbered Ubc9^Low^. We examined if the Ubc9 protein levels correlated with RNA expression in Ubc9^Low^ and Ubc9^HyperLow^ mice. As can be seen in figure 1*i*, the Ubc9^HyperLow^ lysates displayed lower amounts of Ubc9 compared with Ubc9^Low^ lysates. In line with this, the global SUMO-1 and SUMO2/3 profiles of DSS-7 mice were also altered (figure 1*j*,*k*). We also checked the role of various deSUMOylases in the global reduction of SUMOylation in DSS mice. As can be seen in the electronic supplementary material, figure S1, except for SENP7 expression of none of the other SENPs were altered in DSS animals. A mild upregulation of free SUMO2/3 but not SUMO1 was seen in the global SUMOylation blots of DSS-treated animals (figure 1*j*,*k*). To investigate if Ubc9 levels were mainly affected in epithelial cells, gene expression of epithelial cells isolated from the colon was analysed. Clearly, epithelial lysates of DSS-7 mice displayed much lower Ubc9 expression than that of control mice (figure 1*l*). Furthermore, IHC also revealed expression of Ubc9 protein in the epithelial villi near to the nucleus in control mice but much less in DSS-7 mice (figure 1*m*). In line with this, the disease severity index (goblet cell loss, ulcers, epithelial erosions, neutrophil infiltration, etc.), as calculated by a blinded pathologist, also revealed that DSS-7 mice with hyperlow Ubc9 were more severely affected compared with other mice (figure 1*n*). On the basis of these data, we hypothesized that downregulation of Ubc9 may be an important prerequisite for the onset of inflammation.

### 3.2. Ubc9 modulates expression of inflammatory genes in colonic epithelium

**Figure 2.**
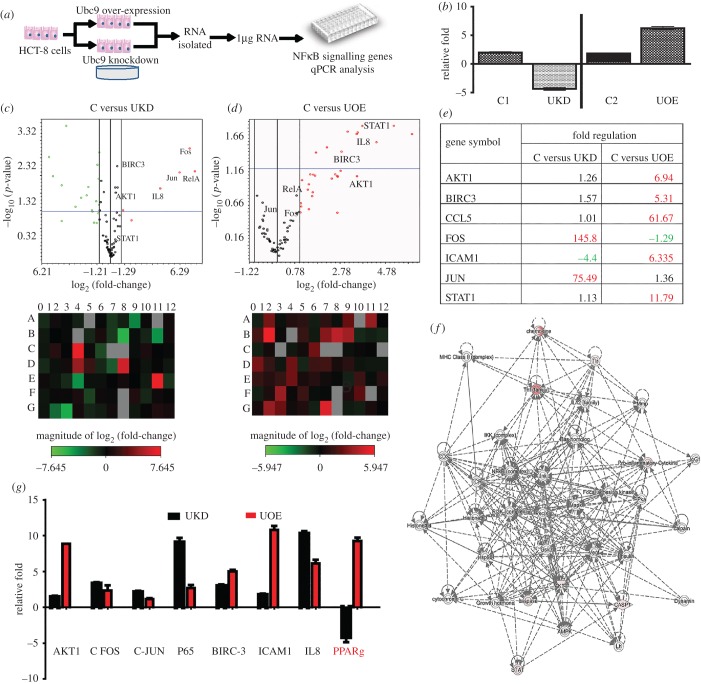
SUMO enzyme Ubc9 controls the expression of inflammatory pathway genes in intestinal epithelial cells. (*a*) Schematic of steps involved in analysis of gene expression in HCT-8 cells using array-based qPCR. (*b*) Validation of Ubc9 expression by qPCR in control, Ubc9 knockdown (UKD) using Ubc9-siRNA and Ubc9 overexpression (UOE) samples. The qPCR array experiment was done in triplicate and the data were analysed as volcano plots (*c*,*d*)(i) and heat maps (*c*,*d*)(ii). The analysis was done as control versus UKD or control versus UOE as represented in (*c*) and (*d*), respectively. In volcano pots, horizontal axis shows fold change (log_2_) and vertical axis represents the statistically significantly dysregulated genes (−log_10_ scale; *p* < 0.05, blue line). (*e*) Table showing the fold change of various significant genes from qPCR array, altered in UKD and UOE samples compared with control. (*f*) Gene networking by IPA reveals Akt1-dependent IPA functional network in UOE sample. Each line represents a direct interaction/regulation, while dotted line indicates indirect interaction/regulation. (*g*) Validation of qPCR array data for indicated genes using in-house oligomers.

To test SUMOylation-dependent modulation of inflammation, we used RNAi-mediated downregulation of Ubc9 in cultured epithelial cells (HCT-8) as shown in figure 2*a*. Inflammation-related gene expression analysis was carried out using qPCR array (SAB Bioscience, USA) for 84 different NFκB signalling pathway genes. As seen in figure 2*b*, compared with the control siRNA-treated cells (si_control cells, or C1 in figure 2*b*), about fourfold to fivefold downregulation of Ubc9 was seen in Ubc9-specific siRNA-treated cells (Ubc knockdown (UKD) cells). The qPCR data revealed a dramatic alteration of inflammatory genes such that genes encoding pro-inflammatory regulators RelA, cFos, cJun and others displayed significant activation (greater than threefold) (volcano plot, figure 2*c*).

Furthermore, overexpression of Ubc9-encoding plasmids (hereafter UOE cells, compared with C2 in figure 2*b*) resulted in severe dysregulation of several NFκB signalling pathway genes as shown in the heat maps of C versus UOE (figure 2*d*). The volcano plots (figure 2*d*) highlight the nature of chaos in inflammatory pathway genes. Crucial regulators such as Akt1, Birc3, Icam1, Stat1 and others were induced by more than threefold (*p* < 0.05) in UOE cells as shown in the tabular representation (figure 2*e*). Interestingly, level of RelA, cFos and cJun were downregulated in UOE conditions, hinting that level of Ubc9 in epithelium tightly controls expression of inflammatory genes. Based on the differential expression of genes in Ubc9 perturbed conditions, we were able to generate a network of interrelationship of genes highlighting nodes and biological relevance between various regulators using IPA software. The kinase Akt1 occupied a central hub in the inflammatory gene network, having regulatory connections with key regulators including STAT, CASP1, TNF family and chemokines (figure 2*f*). These data revealed that the SUMO pathway could potentially modulate the gene expression of the inflammatory pathway. We further validated our findings by individual qPCR analysis for some of these severely affected genes using in-house primers. PPARγ, a crucial regulator of gut homeostasis, known to be regulated by SUMOylation [13,14], displayed an upregulation in UOE samples, while in UKD samples it was downregulated (figure 2*g*). Together, these observations led us to conclude that perturbation of the SUMOylation machinery can bring forth profound changes in the expression of inflammatory genes in the epithelium.

### 3.3. Extent of SUMO E2 enzyme Ubc9 expression correlates with severity of colitis in mice

**Figure 3.**
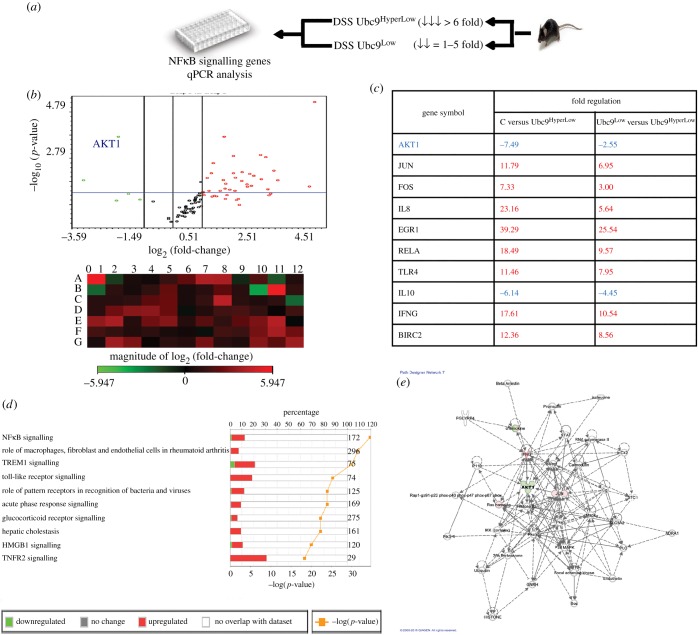
Ubc9 levels correlate with the severity of inflammation in DSS-7 mice. (*a*) Schematic overview of steps involved in gene expression analysis using qPCR in mice. Total RNA was extracted by colonic tissue of DSS-treated animals with hyperlow Ubc9 and low Ubc9 along with the control group. This RNA was processed for qPCR array and the data plotted as volcano plots (*b*, top) display significant inflammatory gene activation in DSS animals. In volcano pots, horizontal axis shows fold change (log_2_) and vertical axis represents the statistically significant dysregulated genes (−log_10_ scale; *p* < 0.05, blue line). Heat maps (*b*, bottom) show pattern of expression of DSS-7-Ubc9^HyperLow^ compared with DSS-7-Ubc9^Low^. (*c*) Table showing the fold change of various significant genes altered in DSS-7 mice samples. (*d*) IPA comparative analysis showing significant signalling altered in DSS-7-Ubc9^HyperLow^ compared with DSS-7-Ubc9^Low^. (*e*) Akt1-dependent IPA functional network.

To extend our understanding of SUMO status and its relevance to the inflammatory network*,* we reverted to our mice model. We analysed gene expression using DSS-7 mice with either low or hyperlow levels of Ubc9, as shown in figure 3*a*. We reasoned that comparison of gene expression in DSS-7-Ubc9^Low^ versus DSS-7-Ubc9^HyperLow^ would highlight the significance of the severe downregulation of Ubc9 and its connection to the inflammatory pathway. Similar to the experiments done in epithelial cells, we carried out expression profiling using an inflammatory gene array (SAB Biosciences, USA) using different groups of animals: DSS-7-Ubc9^Low^, DSS-7-Ubc9^HyperLow^ along with the control untreated group. The experiment was done three times following standard guidelines. The data revealed that several inflammatory markers and other crucial genes of the inflammatory pathway such as cJun, c Fos, TLRs, BIRC2, IL-8, RelA and EGR were induced by greater than threefold (*p* < 0.05) during DSS treatment. A more dramatic activation of inflammatory pathway genes was seen in DSS-7-Ubc9^HyperLow^ versus the DSS-7-Ubc9^Low^ mice (figure 3*b*). More than 20 of 84 genes displayed an induction of 2.5-fold or above (*p* < 0.05) and five of 84 genes displayed 2.5-fold or above downregulation (figure 3*b*) (*p* < 0.05). Intriguingly Akt1, a master kinase which is otherwise not well explored in the context of IBD, was among the downregulated genes in Ubc9^HyperLow^ compared with control mice (−7-fold) and DSS-7-Ubc9^Low^ (−2.5-fold) as shown in figure 3*c*. For master kinases such as Akt1, where even the slightest fluctuations are known to be detrimental, the effect of SUMOylation perturbation seemed intriguing.

We further investigated the RT-PCR data with fold expression and *p*-value threshold (less than 0.05) of genes, with GAPDH as reference, using IPA software, which is a computational tool for the integration and understanding of complex data. Differentially expressed genes from DSS-7-Ubc9^HyperLow^ when compared with DSS-7-Ubc9^Low^ and control mice were overlaid in a database to algorithmically generate a pathway network based on data available in the software. IPA comparative analysis of samples DSS-7-Ubc9^HyperLow^ with respect to DSS-7-Ubc9^low^ revealed involvement of TREM, NFκB, TLR and TNFR2 signalling pathways (figure 3*d*). Based on the differential gene expression in the control and the colitis animals' conditions, we were able to generate a network of interrelationship of genes highlighting nodes and biological relevance between various regulators. Based on the analysis, Akt1 occupied a central hub in the inflammatory gene network, having direct regulatory connections with key regulators including Jun, FAK, IFN-γ, p38 and STAT that have relevance to the field of IBD (figure 3*e*).

### 3.4. Mice with severe intestinal inflammation display a distinct SUMO-1 proteome

**Figure 4.**
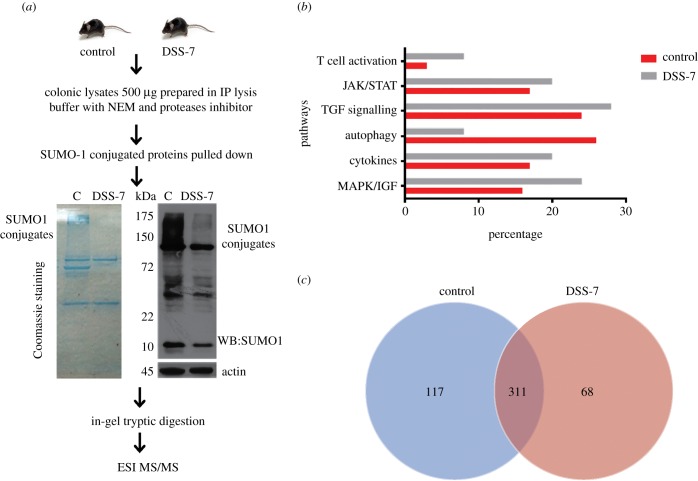
Altered SUMO1 proteome of DSS-7 mice colonic tissue samples. (*a*) Workflow diagram for SUMO1 proteomic analysis involving pull down of mice colonic pooled protein lysates (*n* = 2). Coomassie staining showing SUMO-1 immunoprecipitation (left panel) and SUMO1 immunoblots of control and DSS-7 mice samples with loading control (right panel). Five hundred micrograms of protein were used for anti-SUMO1 IP and samples were prepared using in-gel digestion for MS analysis. (*b*) Relative abundance of various signalling pathways based on mass-spectrometric data from DSS-7 mice compared with control mice. (*c*) Venn diagram displaying the number of individual proteins identified in control and DSS-7 mice samples. Venn diagram was developed using Scaffold software (v. 4.4.8, Proteome Software Inc.).

Next, we set forth to determine the intestinal epithelial SUMOylome. Here, we were interested in understanding the colitis-specific SUMOylome in intestinal inflammation *in vivo*. As SUMO-1 participates in both SUMO-1 and in SUMO2/3-conjugated proteins, we restricted our analysis to the SUMO1-conjugated proteome (hereafter called SUMO-1 proteome). Lysates from control and DSS-7 animals were immunoprecipitated using anti-SUMO-1 antibodies (figure 4*a*) and global repression of SUMO-1 conjugates was clearly seen in DSS-7 mice samples (DSS-7-Ubc9^HyperLow^). The lysates were resolved by SDS–PAGE followed by in-gel tryptic digestion. These samples were subjected to ESI MS/MS using the ABSCIEX 5600 machine (details in Material and methods). The data files were transformed into peptide maps using offline software ProteinPilot and MASCOT. Using the online Panther tool [15], we identified proteins present in various categories. SUMO-1 and SUMO-2 were identified and thus acted as the positive control for our experiment. Several interesting proteins among these included: (i) inflammatory pathways mediated by cytokines, (ii) MAPK/IGF, (iii) JAK/STAT, (iv) T-cell activation and (v) TGFβ signalling were upregulated in DSS-7 compared with control mice as shown in the pathway analysis graph (figure 4*b*). By contrast, proteins involved in autophagy were seen to be more abundant in control samples (figure 4*b*). We observed that several key regulators including Akt1 kinase and BIRC3 were present only in control samples but not in DSS-7 animals, implying lower abundance of their SUMOylated forms in colitis-associated epithelium. The electronic supplementary material, table S1 represents a list of important identified proteins with SUMO-binding motif and (SBM) and SUMO-interacting motif (SIM) in the proteins identified by using GPS SBM 1.0 [16]. Proteins such as NCoR1 [14], RORγ [17], STAT1 [18] and Akt1 [19] have been reported to undergo SUMOylation. Among the identified proteins, several have also been previously reported in bowel disease [20] such as: STAT1 [18], Nod2 [21], ATG9B [22], IRAK1 [23], IL-17R [2], RORγ [17] and NCoR1 [14]. We further analysed our data using Scaffold (v. 4.4.8, Proteome Software Inc.), which uses the peptide prophet and protein prophet algorithms for stringent data analysis. Numbers of different proteins identified in control compared with DSS-7 mice are represented in the Venn diagram (figure 4*c*). Our analysis revealed a large number of proteins, 428 in the control group compared with 379 DSS-7 mice. Among these, 117 proteins exclusively found in control mice were subjected to IPA signalling analysis. These were seen to be associated with thiosulfate disproportion III pathway, granzyme B signalling and ATM signalling (electronic supplementary material, figure S2*a*). Sixty-eight proteins were exclusively found in DSS-7 samples. These were proteins known to be involved in dendritic cell maturation, NRF2 oxidative stress response, hepatic fibrosis and the thioredoxin pathway (electronic supplementary material, figure S2*b*). Moreover, when DSS-7 were compared with control samples, pathways such as EIF2 signalling, mitochondrial dysfunction, eIF4/S6K signalling, unfolded proteins response and viral entry pathways were identified (electronic supplementary material, figure S2*c*). Some of these pathways found from our analysis, such as ILK (integrin-linked kinases) signalling, that are associated with cell proliferation, migration and adhesion [24], mitochondrial dysfunction and unfolded protein response, are relevant to IBD [25]. Interestingly, Akt1 was also present in control samples, but not in DSS-7 samples. Akt1 being a major mediator of cell signalling, we further investigated SUMOylation-dependent Akt1 levels and activity in mice samples.

**Figure 5.**
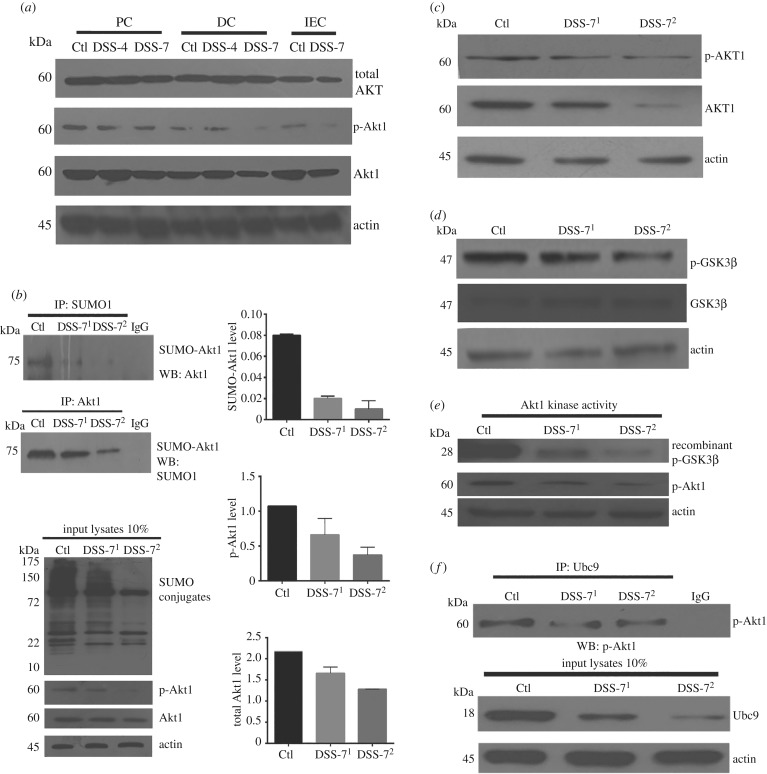
DSS mice display lowered SUMOylated Akt1 and compromised kinase activity. (*a*) Immunoblots of total AKT, Akt1 and pAkt1 (S473) of proximal colon (PC), distal colon (DC) and colonic intestinal epithelium (IEC). (*b*) Various colonic tissue lysates were immunoprecipitated with anti-SUMO1 antibodies (upper panel) and immunoblotted for Akt1 showing reduced SUMO Akt1 in DSS-treated mice and IP with anti-Akt1 antibody and probed for SUMO-1 to detect SUMOylated-Akt1. Ten per cent of the input was loaded as shown in the blot (bottom panel). The ratios of SUMO-Akt1/Akt1 as well as pAkt1/Akt1 are shown based on densitometric values obtained from the IP immunoblots. (*c*) Immunoblots of total Akt1 and phospho-Akt1 (S473) from colonic lysates of mice. (*d*) Immunoblot of phospho-GSK3β (S9) and total GSK3β from mice samples. (*e*) Colonic epithelial lysates from control and DSS-treated animals were added to Akt-specific kinase activity mixture involving recombinant GSK3β (28 kDa), ATP and optimal buffer for 30 min at 30°C. Akt kinase activity was then determined by immunoblotting for pGSK3β. (*f*) Ubc9 and Akt1 interaction was tested using IP of colonic lysates with anti-Ubc9 antibody and probed with anti-phospho-Akt1 antibody, input lysates are represented at the bottom. β-Actin was used as a loading control. In all IP experiments and kinase assays, an equal amount of total protein (500 µg) was used in each category of samples. Mice samples DSS-7^1^ (Ubc9 < sixfold downregulation: Ubc9^Low^) and DSS-7^2^ (Ubc9 > sixfold downregulation: Ubc9^HyperLow^) are compared with control mice samples.

Levels of total Akt (hereafter PAN-Akt) which includes Akt1, Akt2 and Akt3 were examined (figure 5*a*). No significant change was seen in the protein levels of PAN-Akt in DSS-4 and DSS-7 mice in the proximal and distal region of the colon. Interestingly, phosphorylated-Akt1 (active form or pAkt1) increased in DSS-4 mice, while it decreased in DSS-7 mice. This was in line with the reduced levels of Ubc9 in DSS-7 mice. Together, these data reveal that Akt1 and p-AKT1, but not total AKT, were lowered in inflammed intestinal tissues. Next, we carried out IP of mice colonic lysates using SUMO1 antibody. First, SUMO-Akt1 bands displayed higher mobility (approx. 75 kDa) than the unmodified Akt1 (approx. 60 kDa) and were significantly reduced in expression in DSS-treated animals compared with control animals (figure 5*b*). To examine this by an alternative method, samples were immunoprecipitated with Akt1 antibody and probed with SUMO-1 antibody. Similar to above, SUMOylated-Akt1 was seen to be more in control samples compared with those from DSS-7 (figure 5*b*, lower panel). A more pronounced decrease in levels of SUMOyated-Akt1 and Akt1 was seen in DSS-7^HyperLow^ compared with DSS^Low^ or control animals (figure 5*b*). To check proportions of pAkt1 and SUMOylated-Akt1 (or SUMO-Akt1), we loaded exactly 10% of the input used for IP and probed for each of SUMO-1, pAkt1 and Akt1. Precise ratios of pAkt1/SUMO-Akt1 were determined by normalization of densitometry values of the band (figure 5*b*, right panel). Interestingly, the abundance of pAkt1 in samples was 49% of total Akt1 in control lysates, while 36% and 25% of total Akt1 in DSS^Low^ and DSS^HyperLow^ samples, respectively. Notably, SUMO-Akt1 was 3.66% of total Akt1 in control compared with 1.2% and 0.8% in DSS^Low^ and DSS^HyperLow^ samples, respectively. Clearly, the SUMO-Akt1 was most dramatically affected in the DSS^HyperLow^ samples.

To examine the effect of the lowered levels of Akt1 on its function, we probed phosphorylation of GSK3β, a cognate substrate of Akt1. Akt1 is known to catalyse phosphorylation of GSK3β at serine 9 (GSK3βS9). GSK3βS9 phosphorylation was significantly lowered in DSS-7 samples with the levels being lowest in DSS^HyperLow^ samples (figure 5*c*,*d*). To further consolidate this point, we used a non-radioactive Akt kinase activity assay. Phosphorylated Akt1 was immunoprecipitated from tissue lysates followed by *in vitro* measurement of kinase activity on recombinant GSK3β in the presence of ATP. As can be seen in figure 5*e*, both pAkt1 and pGSK3β were lowered in the DSS-treated mice samples. Together, these data suggest that DSS colitis leads to reduced levels of Ubc9, which in turn lead to a lowered level of pAkt1 and its activity.

Literature suggests that SUMOylation of Akt1 is phosphorylation-dependent and promotes its stabilization [19,26]. Mechanistically, it was important to examine if the phosphorylated form of Akt1 was capable of undergoing SUMOylation in the context of intestinal inflammation. We carried out IP experiments using Ubc9 antibody and probed for pAkt1; we observed a strong interaction of phospo-Akt1 with Ubc9, which meant that pAkt1 in intestinal inflammation, similar to earlier studies, was capable of interacting with Ubc9 and undergoing SUMOylation. The interaction was diminished in DSS-treated animals, which may be due to decreased amounts of both pAkt1 and Ubc9 (figure 5*f*). Overall, our data support the role of SUMOylation in DSS-induced colitis in mice and highlights the hypothesis that lowering of Akt1 SUMOylation correlates with severely decreased levels of Ubc9. Given the role of SUMOylation in inflammatory signalling, we next tested the role of SUMOylation in conditions of intestinal inflammation in human intestinal diseases.

### 3.5. Downregulation of Ubc9 and lowered activity of Akt1 in human inflammatory bowel disease samples

**Figure 6.**
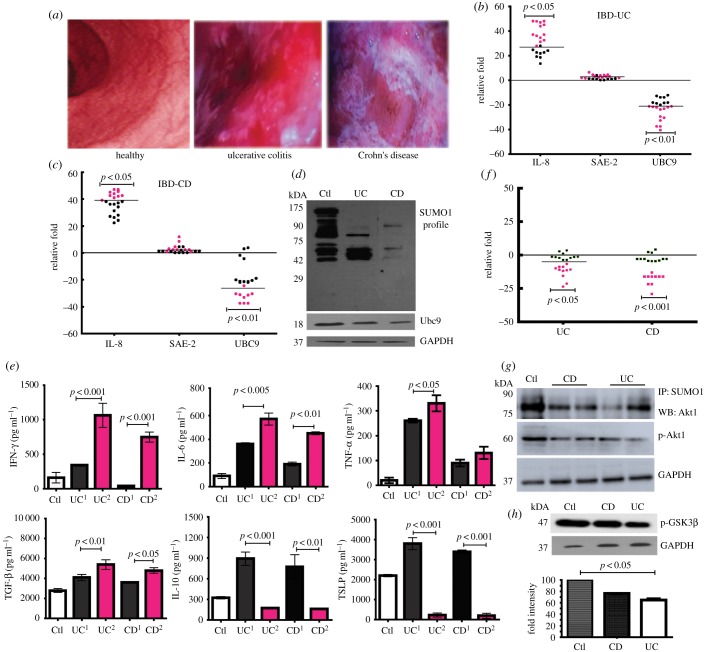
Human CD and UC patient samples display severe downregulation of Ubc9 and Akt1 function. (*a*) Colonoscopy images showing the colon of healthy, UC and CD patients. (*b*,*c*) qPCR analysis of SUMOylation-pathway genes in colonic biopsy samples of human UC (*b*) and CD (*c*) patients normalized to averaged control values (*n* = 22) are plotted as relative fold expression. Each dot represents data from one individual, black dots represent CD^UBC9-Low^ and UC^UBC9-Low^ and red dots represent CD^UBC9-HyperLow^ and UC^UBC9-HyperLow^. (*d*) Immunoblots of SUMO-1 and Ubc9 of patient samples showing overall decrease in SUMO proteome. (*e*) ELISA for IFN-γ, IL-6, TNF-α, TGF-β, IL-10 and TSLP from pooled patient tissue lysates (*n* = 5 in each group) were performed and the specific values as indicated were plotted. UC^1^ and CD^1^ correspond to UC^UBC9Low^ and CD^UBC9Low^, respectively. UC^2^ and CD^2^ correspond to UC^UBC9HyperLow^ and CD^UBC9HyperLow^, respectively. (*f*) qPCR-based fold change expression of Akt1 in human CD and UC patients relative to averaged control values (*n* = 22) are plotted (red dots showing more repression represent CD^UBC9-HyperLow^ and UC^UBC9-HyperLow^ and black dots represent CD^UBC9-Low^ and UC^UBC9-Low^). (*g*) Pooled lysates of control, CD and UC samples (*n* = 5 in each group) were used for IP with anti-SUMO-1 antibody followed by immunoblotting with anti-phospho-Akt1 antibody. (*h*) Akt1-specific kinase activity was assayed by immunoblotting for pGSK3β from pooled lysates (*n* = 5). The densitometry values representing lowered pGSK3β in CD and UC are also represented in the graph (lower panel). For human samples, statistical testing was performed between control and each diseased group using the Mann–Whitney *U*-test (*p*-values as indicated). For qRT, GAPDH and HPRT ertr taken for normalization.

To investigate if these findings are relevant to human IBD patients, colonic biopsy samples were obtained by lower-endoscopic procedure in patients (age group 15–60 years) suffering from UC, CD and control individuals (suspected of IBD but declared negative). The required sample size was calculated to be 22 (statistical power of greater than 80% and α = 5% per group) for each of CD, UC and control groups based on our pilot study. Compared with control individuals, intestines of CD and UC patients displayed inflammation as can be seen by images taken during colonoscopy (figure 6*a*). This was also evident from qPCR IL-8 mRNA expression analysis (figure 6*b*,*c*), which revealed significant upregulation in CD and UC samples. In the case of SUMOylation pathway genes, while the expression of Sae-2 remained more or less unchanged, the expression of Ubc9 displayed drastic repression, between 25- and 50-fold in some cases (red dots, figure 6*b*,*c*) and five- and 25-fold (black dots figure 6*b*,*c*) in others. The Ubc9 protein levels were also reduced in both UC and CD (figure 6*d*). We examined if reduced levels of Ubc9 had an effect on global SUMOylation in these samples. We observed a significant downmodulation of the overall SUMO conjugation in the UC and CD samples (figure 6*d*).

To further understand the role of Ubc9 in the pathophysiology of IBD, we subgrouped the patients with varying levels of Ubc9, such that Ubc9 mildly downregulated samples (between five- and 25-fold) of CD were referred to as CD^UBC9-Low^ and of UC as UC^UBC9-Low^. Similarly, the more severely downregulated Ubc9 (between 25- and 50-fold) in CD samples were referred to as CD^UBC9-HyperLow^ and UC as UC^UBC9-HyperLow^. Interestingly, the CD^UBC9-HyperLow^ and UC^UBC9-HyperLow^ samples displayed a higher IL-8 mRNA compared with CD^UBC9-Low^ and UC^UBC9-Low^, respectively (figure 6*b*,*c*). We also examined the expression of several E3-SUMO enzymes, among which PIAS1 [27] was also downregulated in both UC and CD (data not shown).

To overcome the limitation of sample quantity and to get an averaged essence, we pooled samples (five in each category as explained in Material and methods) and carried out ELISAs of pro-inflammatory cytokines such as IL-6, TNF-α and IFN-γ. Each of these marker cytokines were elevated in the CD and UC samples. Intriguingly, their levels were further elevated in CD^UBC9-HyperLow^ and UC^UBC9-HyperLow^ (figure 6*e*). Levels of anti-inflammatory cytokines including TSLP, IL-10 and TGF-β were lesser in the CD^UBC9-HyperLow^ and UC^UBC9-HyperLow^ compared with CD^UBC9-Low^ and UC^UBC9-Low^ (figure 6*e*). TGFβ was slightly higher in the CD and UC samples, with no major difference between groups with varying Ubc9. This cytokine has anyway been assigned dual roles including pro- and anti-inflammatory activities in the context of the gastrointestinal tract [4].

Next, we wanted to study the correlation of Ubc9 repression with the disease severity in CD and UC patients. The details of the UC and CD patient clinical parameters are shown in table 1. Briefly, four (18.18%) patients had left-sided colitis, and 18 (72.7%) had pancolitis in UC, whereas in CD most (20, 92.9%) had L2 colon disease. Out of all 44 patients in both diseased groups, 26 had mild and 18 had moderate to severe disease activity (17 moderate, one severe). The baseline characteristics of UC and CD patients with disease activity index are shown in table 1. The decrease in expression of UBC-9 was correlated with UCDAI and CDAI in UC and CD patients, respectively. The correlation indicated that the UCDAI and CDAI were maximal in the case of UC ^UBC9-hyperLow^ and CD ^UBC9-hyperLow^ among UC and CD patients, respectively. In the case of UC patients, the UBC9^HyperLow^ category displayed a higher number of patients in relapse when compared with the UBC9^Low^ category. Next, we investigated the status of Ubc9 substrate Akt1 in UC and CD of pooled biopsy samples. As expected, these samples also displayed the same pattern of lowering of Akt1 at RNA levels in UC and CD patients samples (figure 6*f*). Repression was also seen in the case of p-Akt1, and akin to the DSS mice model SUMO-conjugated pools of Akt1 were lower in CD and UC patients as seen by SUMO-1 IP of the protein lysates (figure 6*g*). The status of Akt substrate phospho-GSK3β was also lowered in CD and UC patients compared with controls (figure 6*h*). Together, these data reveal that in IBD patient samples the SUMOylation machinery functions at suboptimal levels and this is accompanied with lowered Akt1 activity and heightened inflammation.

**Table 1.** Baseline characteristics of UC and CD patients along with disease severity correlated with UBC9 level.

	ulcerative colitis characteristics	UBC9^Low^ (*n* = 6)	UBC9^HyperLow^ (*n* = 16)	*p*-value
1	gender (male : female)	4 : 2	10 : 6	0.58
2	age in years (mean ± s.d.)	37.7 ± 14.1	36.4 ± 14.4	0.32
3	duration of disease (mean ± s.d.) in years	2.24 ± 1.05	2.37 ± 1.04	
4	UCDAI at entry (mean ± s.d.)	0.82 ± 0.33	5.48 ± 1.43	0.005
5	disease extent (number of patients)			
	left-sided colitis	2 (33.8%)	2 (12.8%)	
	pancolitis	4 (66.5%)	14 (87.5%)	
6	concomitant medications (number of patients)			
	5-aminosalicylic acid	6 (100%)	12 (75%)	
	steroids	0 (0%)	0 (0%)	
	azathioprine	4 (66.67%)	8 (50%)	
	Crohn's disease characteristics	UBC9^Low^ (*n* = 10)	UBC9^HyperLow^ (*n* = 12)	*p-*value
1	gender (male : female)	6 : 4	10 : 2	0.58
2	age in years (mean ± s.d.)	40.2 ± 4.8	42.4 ± 8.2	
3	duration of disease (mean ± s.d.) in years	2.86 ± 1.23	3.48 ± 1.04	
4	CDAI at entry (mean ± s.d.)	166.34 ± 8.33	315.67 ± 22.43	0.021
5	disease extent (number of patients)			
	L1: terminal ileum	0 (0.0%)	0 (0%)	
	L2: colon	10 (100%)	10 (83.2%)	
	L3: ileocolonic	0 (0.0%)	2 (16.8%)	
6	concomitant medications (number of patients)			
	5-aminosalicylic acid	10 (100%)	8 (66.7%)	
	steroids	0 (0%)	0 (0%)	
	azathioprine	10 (100%)	6 (50%)	

### 3.6. Ubc9 downregulation subprogrammes inflammatory environment via Akt1 signalling in epithelial cells

**Figure 7.**
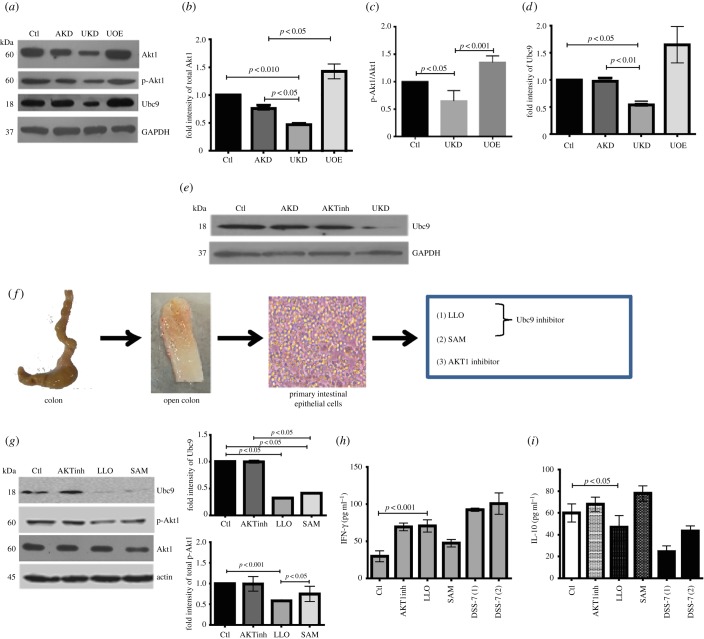
Experimental repression of Ubc9 in primary and cultured epithelial cells downmodulates active Akt1. (*a*) Immunoblots with indicated antibodies from lysates of HCT-8 cells with either Akt1 knockdown using Akt1-siRNA (AKD), Ubc9 knock-down using Ubc9-siRNA (UKD), Ubc9 over-expression using pUbc9 (UOE), along with corresponding control lysates (Ctl). Corresponding densitometric values of total Akt1 (*b*), ratios of pAkt1/Akt1 (*c*) and Ubc9 (*d*) were also represented as bar graphs. (*e*) Immunoblots of Ubc9 from HCT-8 cells treated specifically with Akt1 kinase inhibitor (AKTinh), siRNA-for Akt1 (AKD), UKD or left untreated (Ctl). (*f*) Schematic overview of steps involved in *ex vivo* culturing of PIECs from mice colon (actual picture of epithelial culture represented here). (*g*) Immunoblots of Ubc9, p-Akt1 and total Akt1 from lysates of PIEC treated with the indicated inhibitors (AKTinh, Akt1 inhibitor; LLO, listeriolysin; SAM, *S*-adenosyl methionine). Right panel: densitometric analysis of immunoblots. (*h*,*i*) ELISA for IFN-γ and IL-10 of supernatant of PIECs treated as indicated. Supernatant of DSS-7 mice primary epithelial cells were used as positive controls for inflammation.

Lowering of Ubc9 in DSS mice and IBD patient samples resulted in increased disease severity indices and this led us to ask whether Ubc9 alteration is the basis for the exacerbated colitis or merely a consequence of the underlying disorder. Therefore, we next set forth to understand the interplay of Akt1 and Ubc9 in detail using *in vitro* and *ex vivo* models. In Ubc9 knocked-down HCT-8 cells, we observed a significant lowering of total Akt1 and pAkt1 (figure 7*a*–*c*). Interestingly, Ubc9 upregulation resulted in an increase in the Akt1 protein and pAkt1 levels (figure 7*a*), thus revealing a possible direct upstream effect of Ubc9 on Akt1 expression. The densitometry of the blots shows that these changes were significant (figure 7*b*–*d*). Inhibition of Akt1 by Akt-specific inhibitor or Akt1-siRNA in HCT-8 cells did not result in significant changes in levels of Ubc9 (figure 7*e*), thus suggesting that Ubc9 controls Akt1 expression and not vice versa.

To test if the Ubc9-mediated regulation of Akt1 was not an effect restricted only to cell lines, we analysed gene expression in primary intestinal epithelial cells (PIECs) isolated from mice colon (figure 7*f*). As PIECs are recalcitrant to genetic manipulations, we used chemical agents to inhibit Ubc9 [9,28] and Akt1. As can been seen in figure 7*g*, inhibition of Ubc9 by SAM or LLO resulted in concomitant loss of p-Akt1 levels. On the other hand, inhibition of Akt1 with a chemical inhibitor (Akt inhibitor VIII trifluoroacetate salt hydrate, Sigma) did not lead to any detectable reduction in levels of Ubc9. The densitometric analysis of the blots is also plotted (figure 7*g* right-hand panel). Moreover, the culture supernatants of PIECs inhibited for p-Akt1 showed increased levels of IFN-γ, thus pointing towards an increase in pro-inflammatory signalling (figure 7*h*). Similar to these, LLO and SAM-treated PIECs also displayed an increase in IFN-γ levels. In the case of LLO treatment, there was also a decrease in levels of anti-inflammatory cytokine IL-10 (figure 7*i*). The cytokine levels were also checked in DSS-7-treated mice epithelial cells for comparison, in which IFN-γ was secreted more and IL-10 was secreted less compared with cells from control animals. Together, these data suggest that Ubc9 modulates expression of inflammatory cytokines via Akt1.

### 3.7. Epithelial wound healing is linked to Akt1 SUMOylation

**Figure 8.**
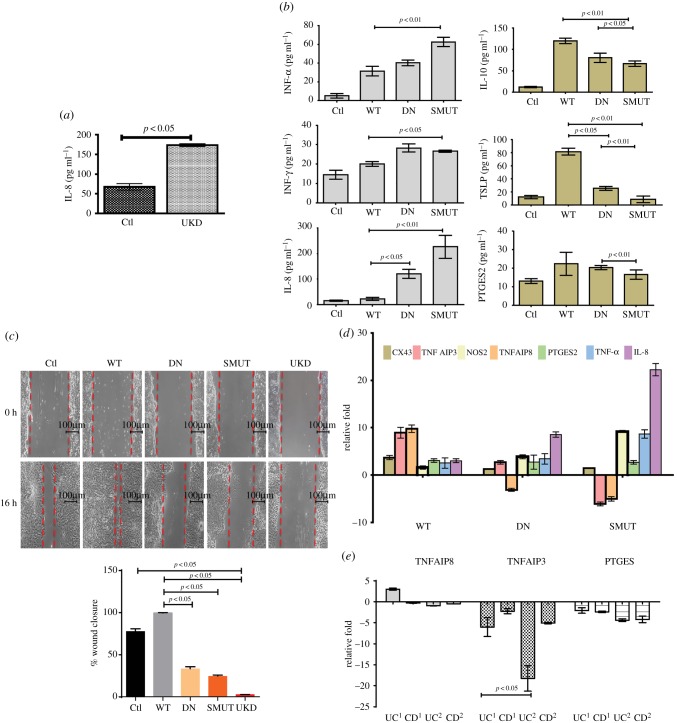
SUMOylation-deficient Akt1 exacerbates inflammation accompanied with impaired wound healing. (*a*) Values of IL-8 ELISA of supernatants from HCT-8 cells knocked-down for Ubc9 (UKD) or untreated cells. (*b*) ELISA of indicated cytokine with supernatants from HCT-8 cells transfected with WT-Akt1, DN-Akt1 (kinase dead) and SMUT-Akt1 (SUMO-deficient) plasmids. (*c*) Cell migration assessed by wound-healing assay in untreated (Ctl) or WT-Akt1, kinase dead Akt1 (DN) and SUMOylation-deficient Akt1 encoding plasmid transfected HCT-8 cells and Ubc9 knocked-down cells (UKD). The wound closure scores with statistics are plotted (lower panel), which show the compromised healing seen in SMUT and UKD samples. (*d*) qRT-PCR gene expression of wound-healing and inflammatory markers in WT-Akt1, DN-Akt-1 (kinase dead) and SMUT-Akt1 (SUMO-deficient) samples compared with control. (*e*) qRT-PCR gene expression of TNFAIP3, TNFAIP8 and PTGES in human IBD patient samples. UC^1^ and CD^1^ correspond to UC^UBC9-Low^ and CD^UBC9-Low^, respectively. UC^2^ and CD^2^ correspond to UC^UBC9-HyperLow^ and CD^UBC9-HyperLow^, respectively. For qRT, GAPDH and HPRT were taken for normalization.

To examine if Ubc9 and Akt1 crosstalk leading to heightened inflammation was due to reduced healing of epithelium, we carried out cytokine assays and wound-healing assays in epithelial cells. In these assays, supernatant of UKD samples in HCT-8 cells resulted in increased secretion of IL-8 (figure 8*a*). Similar results were also obtained when HeLA cells, treated with Ubc9-specific siRNA and stimulated with IL-6, were examined for inflammatory cytokines compared with control cells (data not shown). In line with this result, expression of a SUMOylation-deficient K276R mutant of Akt1 (hereafter SMUT-Akt1) displayed higher levels of pro-inflammatory cytokines. Protein levels of pro-inflammatory cytokines IL-8, TNF-α and IFN-γ were seen to be higher in supernatants of cells overexpressing SMUT-Akt1 compared with those from wild-type (WT) or kinase-dead Akt1 (DN). Levels of anti-inflammatory cytokines TSLP and IL-10 were blunted in SMUT-Akt1 transfected cells (figure 8*b*). As the Akt1/β-catenin pathway is strongly connected to wound healing and epithelial proliferation [29], we reasoned that the observed Akt1-dependent inflammatory signalling could be due to failure of wound healing. We carried out wound-healing assays [12] using HCT-8 cells. WT Akt1 transfection leads to significant healing compared with the kinase dead DN-Akt1 or SMUT-Akt1 (figure 8*c*). Healing was also seen to be less in UKD cells (figure 8*c*). Interestingly, SMUT-Akt1-expressing cells were most severely affected. This was also evident from the wound closure analysis (figure 8*c*, bottom panel). Expression of crucial wound-healing markers TNFIAP3 and TNFIAP8 increased (up to 10-fold) in WT-Akt1. TNFIAP3 and TNFIAP8 were downregulated in SMUT-Akt1 samples (figure 8*d*). The expression of NOS-2 was upregulated in SMUT-Akt1. No significant change was seen in the expression of Cx43 and PTGES2 genes. In line with this expression, TNFAIP3 and TNFAIP8 were severely downregulated in UC^UBC9-hyperLow^ human patient biopsy samples. Level of PTGES2 on the other hand was downregulated in both UC and CD UBC9-^HyperLow^ patient samples compared with UC and CD UBC9^Low^ samples (figure 8*e*). Together, these data reveal that Ubc9-dependent lowering of Akt1, particularly SUMOylated-Akt1, may be responsible for failure of wound healing which itself may be promoting pro-inflammatory mechanisms in epithelium. Overall, our results highlight that these changes are detrimental to functioning of inflammatory regulators and production of key cytokines, events that are important for intestinal inflammation.

## 4. Discussion

In this work, we have investigated the importance of SUMOylation and its connection to IBD. We demonstrated that SUMOylation alteration perhaps assumes a central role in subprogramming molecular pathways when cells transit from a healthy state to an inflamed state. Although SUMOylation has not been explored at all in IBD, it is not surprising that SUMOylation is important, given it has been an emerging paradigm in the case of inflammatory diseases and immune biology [10,30]. The causative reason for the observed SUMOylation alteration was downregulation of the sole SUMO E2 conjugating enzyme, Ubc9. This enzyme, being the only E2 enzyme, is seen to be sufficient to regulate the overall SUMO status as seen in many instances including a mutant cell model of colon cancer, brain astrocytes, KRAS and invasion of colonic cells by gut pathogens like *Salmonella* and others [8,31–34]. Similarly, in rat brain cells, SUMOylation was required for the suppression of inflammatory responses by LXRs in IFN-γ-stimulated astrocytes [35]. Thus, there are numerous examples of SUMOylation-dependent control of inflammation including those involving SENPs [30]. The downregulation of Ubc9 and global SUMOylation leads to an alteration in the SUMOylome which potentially modulates the cellular proteome leading to dramatic signalling and transcriptional subprogramming [36,37]. PTM machineries acting as molecular switches and governing cellular transcriptional programmes have been recently reported [38]. Control of inflammatory regulators PIAS1, NFκB and JunB is already known [39]. These regulatory factors are equipped with abilities to bring forth rapid and reversible changes in a cell in a context-dependent manner.

Our experiments in the DSS mice model revealed Ubc9 expression alteration begins even before the onset of discernible symptoms of inflammation. Intriguingly, Ubc9 downregulation was discernible mainly in the proximal colon initially (day 3); possibly this might be tissue level signalling that later transduces to the distal colon in terms of inflammation, as on day 7 Ubc9 decrease was observed in distal colon as well. In mice as well as in human patient samples, the disease severity, as may be seen by cytokine profiling or disease severity index, was more pronounced depending on the extent of Ubc9 downregulation.

The epithelium is known to regulate the dendritic cell and other immune cell function by secreting a battery of cytokines such as thymic stromal lymphopoietin (TSLP), transforming growth factor-β (TGF-β) and prostaglandin E2 [40,41]. Epithelial Hassall's corpuscles of the thymus secrete TSLP, where it activates myeloid dendritic cells, a process that promotes the proliferation of regulatory T cells [42]. TSLP expression in some mucosal surfaces have been reported to be NFκB-dependent [43]. In line with this, these patients displayed a higher levels of IFN-γ, a pro-inflammatory cytokine known to be secreted by dendritic cells and other immune effectors in response to the epithelial signals [40,41]. Intestinal epithelial cells secrete TSLP to condition the non-inflammatory status of dendritic cells, and they later secrete IL-10 and IL-6 which programmes a TH_2_ non-inflammatory response [43]. Also, TGF-β that is known to negatively regulate the NFκB-dependent genes was found to be lower in the patient samples with UBC9^HyperLow^. Contrary to this, pro-inflammatory cytokines such as IFN-γ, TNF-α and IL-6 were considerably higher in CD^UBC9-HyperLow^ and UC^UBC9-HyperLow^ compared with the control group (*p* < 0.05). This implied that in the patients with lowered Ubc9 and SUMOylation machinery, the conditions were more favourable for inflammation.

The DSS-colitis mice displayed a distinct SUMOylome compared with control animals. This may be due to several factors, the primary one being downregulation of Ubc9. The role of E3 ligases and SENPs may have either additive or synergistic effect, which cannot be ruled out.

Among the affected candidates, we saw a large group of factors that were transcriptional regulators, inflammatory mediators, kinases (such as Akt1), immune regulators and vesicular transport system members. This gives us an idea of the scale at which altered SUMOylation machinery can affect the cell. A computational analysis of genes known to be important for IBD reveals that several among them have potential SUMO conjugation sites and/or SIMs (electronic supplementary material, table S2)*.* Involvement of Akt1 in colitis as revealed by our study is one such example. It is surprising that this important kinase has not been well explored in the pathophysiology of IBD. Total AKT (Akt1, Akt2 and Akt3) increased during development of various cancers, but in contrast, Akt1 levels were seen to be diminished [44]. Thus, the various isoforms of AKT are non-redundant and have context-dependent functions [45,46]. Recently, several groups have reported the role of SUMOylation in controlling Akt1 function [19,26]. In our case, we saw lowering of levels of Akt1 gene transcripts, total Akt and phospho-Akt1. These changes were accompanied with compromised activity of Akt1 in cells with lowered Ubc9. This is in line with a report by Lin *et al.* [19] who demonstrated that Akt1 SUMOylation enhances its own stability and the status of global cellular SUMOylation. Deleterious effects of Akt1 lowering were reported by Ding *et al.* [47], who observed repressed levels of pAkt1 (S473) kinase in inflamed Ubc9-defecient Treg cells. Similarly, ablation of Akt1, but not Akt2, promoted DSS-induced IBD in mice [48]. ERK signalling activation and epithelial and mesenchymal transition (EMT) is induced upon Akt1 downregulation. EMT is involved in chronic inflammation during IBD [49]. The connection of inflammation and Akt1 substrate GSK3β was also seen in our work. GSK3β signalling is a very complex phenomenon and its precise connection to IBD will be an interesting avenue for future investigations. IBD pathophysiology involves cumulative effects of epithelial inflammatory signalling as well as wound-healing mechanisms. We also observed that both SMUT-Akt1 cells and those that are knocked-down for Ubc9 displayed compromised wound healing. A positive role of Akt pathway in wound healing and cell proliferation has been well reported, which supports our results [50–53]. During IBD, intestinal tissue actively engages in wound-healing mechanisms. Failure of wound healing, as seen in our experiments in the case of CD^UBC9-HyperLow^ or UC^UBC9-HyperLow^, may activate further inflammation and result in a vicious cycle leading to disease severity.

**Figure 9.**
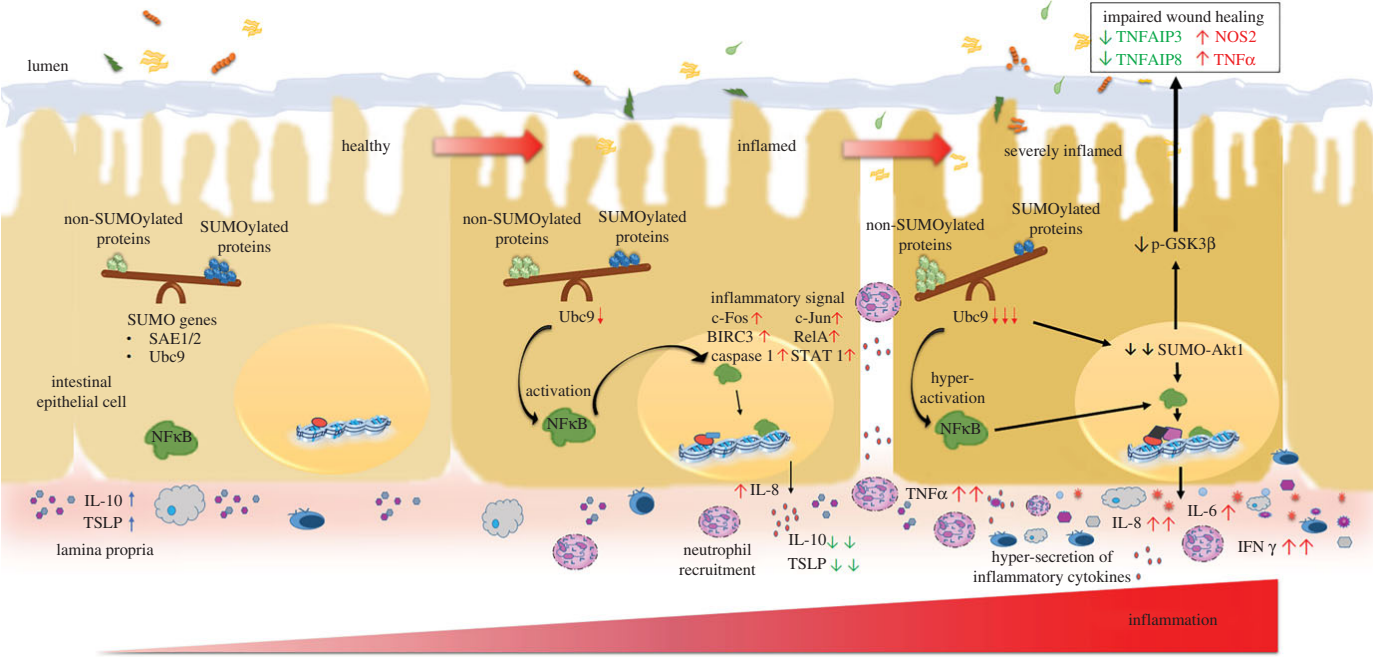
The SUMO pathway plays a crucial role in IBD: model representation of importance of SUMOylation in intestinal health and disease. Steady-state events of the healthy epithelium are represented on the left. A fine balance between SUMOylated and non-SUMOylated proteins is maintained. During the onset of disease, due to downregulation of the sole E2 SUMO conjugating enzyme Ubc9, the balance of SUMOylated and non-SUMOylated proteins is tilted as shown in the see-saw. This activates the NFκB signalling pathway via master signalling regulator Akt1. These changes results in upregulation of transcriptional factors like cFOS, cJUN, RelA, STAT1, BIRC3 (upward arrows). Release of chemokine IL-8/CXCL8 for neutrophil recruitment and lower levels of anti-inflammatory cytokine IL-10 and TSLP further aggravate the condition (severely inflamed cell on the right side). These events leads to compromised expression of wound-healing markers TNFAIP3 and TNFAIP8 (downward arrows) leading to impaired wound healing.

Our work highlights the role of SUMOylation in multiple points of the inflammatory pathway (figure 9) and thus raises the potential of SUMOylation as a single point that may be targeted for fine-tuning the inflammatory circuit for therapeutic treatment of autoimmune disorders. As SUMOylation is the common switch for the regulators that control intestinal inflammation and disease, we postulate that this novel theme may be taken as a new lead for therapeutic intervention for IBD.

## Supplementary Material

Supplementary material Salman et al

## Data accessibility

All the data, code and material concerning the work are will be made publically available and are already provided within the manuscript. These are either part of the provided main dataset or the electronic supplementary material.

## References

[1] Franke A et al. 2010 Genome-wide meta-analysis increases to 71 the number of confirmed Crohn's disease susceptibility loci. Nat. Genet. 42, 1118–1125. (doi:10.1038/ng.717) 21102463 10.1038/ng.717 PMC3299551

[2] Anderson CA et al. 2011 Meta-analysis identifies 29 additional ulcerative colitis risk loci, increasing the number of confirmed associations to 47. Nat. Genet. 43, 246–252. (doi:10.1038/ng.764) 21297633 10.1038/ng.764 PMC3084597

[3] Hooper LV, Littman DR, Macpherson AJ 2012 Interactions between the microbiota and the immune system. Science 336, 1268–1273. (doi:10.1126/science.1223490) 22674334 10.1126/science.1223490 PMC4420145

[4] Yu LC-H, Wang J-T, Wei S-C, Ni Y-H 2012 Host–microbial interactions and regulation of intestinal epithelial barrier function: from physiology to pathology. World J. Gastrointest. Pathophysiol. 3, 27–43. (doi:10.4291/wjgp.v3.i1.27) 22368784 10.4291/wjgp.v3.i1.27 PMC3284523

[5] Ehrentraut SF, Colgan SP 2012 Implications of protein post-translational modifications in IBD. Inflamm. Bowel Dis. 18, 1378–1388. (doi:10.1002/ibd.22859) 22223542 10.1002/ibd.22859 PMC3378042

[6] Hay RT 2005 SUMO. Mol. Cell 18, 1–12. (doi:10.1016/j.molcel.2005.03.012) 15808504 10.1016/j.molcel.2005.03.012

[7] Flotho A, Melchior F 2013 Sumoylation: a regulatory protein modification in health and disease. Annu. Rev. Biochem. 82, 357–385. (doi:10.1146/annurev-biochem-061909-093311) 23746258 10.1146/annurev-biochem-061909-093311

[8] Verma S, Mohapatra G, Ahmad SM, Rana S, Jain S, Khalsa JK, Srikanth CV 2015 Salmonella engages host microRNAs to modulate SUMOylation: a new arsenal for intracellular survival. Mol. Cell. Biol. 35, 2932–2946. (doi:10.1128/MCB.00397-15) 26100020 10.1128/MCB.00397-15 PMC4525320

[9] Ribet D et al. 2010 *Listeria monocytogenes* impairs SUMOylation for efficient infection. Nature 464, 1192–1195. (doi:10.1038/nature08963) 20414307 10.1038/nature08963 PMC3627292

[10] Mabb AM, Miyamoto S 2007 SUMO and NF-κB ties. Cell. Mol. Life Sci. 64, 1979–1996. (doi:10.1007/s00018-007-7005-2) 17530464 10.1007/s00018-007-7005-2 PMC11136396

[11] Keller A, Nesvizhskii AI, Kolker E, Aebersold R. 2002 Empirical statistical model to estimate the accuracy of peptide identifications made by MS/MS and database search. Anal. Chem. 74, 5383–5392. 12403597 10.1021/ac025747h

[12] Li R, Wei J, Jiang C, Liu D, Deng L, Zhang K, Wang P 2013 Akt SUMOylation regulates cell proliferation and tumorigenesis. Cancer Res. 73, 5742–5753. (doi:10.1158/0008-5472.CAN-13-0538) 23884910 10.1158/0008-5472.CAN-13-0538

[13] Lu J, Matunis MJ 2013 A mediator methylation mystery: JMJD1C demethylates MDC1 to regulate DNA repair. Nat. Struct. Mol. Biol. 20, 1346–1348. (doi:10.1038/nsmb.2729) 24304913 10.1038/nsmb.2729 PMC4131721

[14] Pascual G et al. 2005 A SUMOylation-dependent pathway mediates transrepression of inflammatory response genes by PPAR-γ. Nature 437, 759–763. (doi:10.1038/nature03988) 16127449 10.1038/nature03988 PMC1464798

[15] Thomas PD, Campbell MJ, Kejariwal A, Mi H, Karlak B, Daverman R, Diemer K, Muruganujan A, Narechania A. 2003 PANTHER: a library of protein families and subfamilies indexed by function. *Genome Res*. **13,** 2129–2141. (doi:10.1101/gr.772403) 10.1101/gr.772403 PMC403709 12952881

[16] Zhao Q, Xie Y, Zheng Y, Jiang S, Liu W, Mu W, Zhao Y, Xue Y, Ren J. 2014 GPS-SUMO: a tool for the prediction of sumoylation sites and SUMO–interaction motifs. *Nucleic Acids Res*. **42,** W325–W330. (doi:10.1093/nar/gku383) 10.1093/nar/gku383 PMC4086084 24880689

[17] Cook DN, Kang HS, Jetten AM 2015 Retinoic acid-related orphan receptors (RORs): regulatory functions in immunity, development, circadian rhythm, and metabolism. Nucl. Recept. Res. 2, 101185. (doi:10.11131/2015/101185) 10.11131/2015/101185 PMC4750502 26878025

[18] Li Y, de Haar C, Peppelenbosch MP, van der Woude CJ 2012 New insights into the role of STAT3 in IBD. Inflamm. Bowel Dis. 18, 1177–1183. (doi:10.1002/ibd.21884) 21994179 10.1002/ibd.21884

[19] Lin CH, Liu SY, Lee EHY 2016 SUMO modification of Akt regulates global SUMOylation and substrate SUMOylation specificity through Akt phosphorylation of Ubc9 and SUMO1. Oncogene 35, 595–607. (doi:10.1038/onc.2015.115) 25867063 10.1038/onc.2015.115

[20] Pekow J et al. 2013 Gene signature distinguishes patients with chronic ulcerative colitis harboring remote neoplastic lesions. Inflamm. Bowel Dis. 19, 461–470. (doi:10.1097/MIB.0b013e3182802bac) 23388545 10.1097/MIB.0b013e3182802bac PMC3836269

[21] Cho JH, Abraham C 2007 Inflammatory bowel disease genetics: Nod2. Annu. Rev. Med. 58, 401–416. (doi:10.1146/annurev.med.58.061705.145024) 16987083 10.1146/annurev.med.58.061705.145024

[22] Huett A, Goel G, Xavier RJ 2010 A systems biology viewpoint on autophagy in health and disease. Curr. Opin. Gastroenterol. 26, 302–309. (doi:10.1097/MOG.0b013e32833ae2ed) 20571384 10.1097/MOG.0b013e32833ae2ed

[23] Weersma RK, Oostenbrug LE, Nolte IM, Van Der Steege G, Oosterom E, Van Dullemen HM, Kleibeuker JH, Dijkstra G 2007 Association of interleukin-1 receptor-associated kinase M (IRAK-M) and inflammatory bowel diseases. Scand. J. Gastroenterol. 42, 827–833. (doi:10.1080/00365520601114024) 17558906 10.1080/00365520601114024

[24] Yen C-F, Wang H-S, Lee C-L, Liao S-K 2014 Roles of integrin-linked kinase in cell signaling and its perspectives as a therapeutic target. Gynecol. Minim. Invasive Ther. 3, 67–72. (doi:10.1016/j.gmit.2014.06.002)

[25] Novak EA, Mollen KP 2015 Mitochondrial dysfunction in inflammatory bowel disease. Front. Cell Dev. Biol. 3, 62 (doi:10.3389/fcell.2015.00062) 26484345 10.3389/fcell.2015.00062 PMC4589667

[26] de la Cruz-Herrera CF, Campagna M, Lang V, del Carmen González-Santamaría J, Marcos-Villar L, Rodríguez MS, Vidal A, Collado M, Rivas C 2015 SUMOylation regulates AKT1 activity. Oncogene 34, 1442–1450. (doi:10.1038/onc.2014.48) 24704831 10.1038/onc.2014.48

[27] Liu B, Shuai K 2008 Targeting the PIAS1 SUMO ligase pathway to control inflammation. Trends Pharmacol. Sci. 29, 505–509. (doi:10.1016/j.tips.2008.07.008) 18755518 10.1016/j.tips.2008.07.008 PMC2701905

[28] Tomasi ML, Tomasi I, Ramani K, Pascale RM, Xu J, Giordano P, Mato JM, Lu SC 2012 S-adenosyl methionine regulates ubiquitin-conjugating enzyme 9 protein expression and sumoylation in murine liver and human cancers. Hepatology 56, 982–993. (doi:10.1002/hep.25701) 22407595 10.1002/hep.25701 PMC3378793

[29] Nava P et al. 2010 Interferon-gamma regulates intestinal epithelial homeostasis through converging beta-catenin signaling pathways. Immunity 32, 392–402. (doi:10.1016/j.immuni.2010.03.001) 20303298 10.1016/j.immuni.2010.03.001 PMC2859189

[30] Huang W et al. 2011 Coronin 2A mediates actin-dependent de-repression of inflammatory response genes. Nature 470, 414–418. (doi:10.1038/nature09703) 21331046 10.1038/nature09703 PMC3464905

[31] Fritah S, Lhocine N, Golebiowski F, Mounier J, Andrieux A, Jouvion G, Hay RT, Sansonetti P, Dejean A 2014 Sumoylation controls host anti-bacterial response to the gut invasive pathogen *Shigella flexneri*. EMBO Rep. 15, 965–972. (doi:10.15252/embr.201338386) 25097252 10.15252/embr.201338386 PMC4198040

[32] Choi SJ et al. 2006 Negative modulation of RXRalpha transcriptional activity by small ubiquitin-related modifier (SUMO) modification and its reversal by SUMO-specific protease SUSP1. J. Biol. Chem. 281, 30 669–30 677. (doi:10.1074/jbc.M604033200) 10.1074/jbc.M604033200 16912044

[33] Akar CA, Feinstein DL 2009 Modulation of inducible nitric oxide synthase expression by sumoylation. J. Neuroinflammation 6, 12 (doi:10.1186/1742-2094-6-12) 19323834 10.1186/1742-2094-6-12 PMC2667488

[34] Yu B et al. 2015 Oncogenesis driven by the Ras/Raf pathway requires the SUMO E2 ligase Ubc9. Proc. Natl Acad. Sci. USA 112, E1724–E1733. (doi:10.1073/pnas.1415569112) 25805818 10.1073/pnas.1415569112 PMC4394293

[35] Lee JH, Park SM, Kim OS, Lee CS, Woo JH, Park SJ, Joe E, Jou I 2009 Differential SUMOylation of LXRalpha and LXRbeta mediates transrepression of STAT1 inflammatory signaling in IFN-γ-stimulated brain astrocytes. Mol. Cell 35, 806–817. (doi:10.1016/j.molcel.2009.07.021) 19782030 10.1016/j.molcel.2009.07.021

[36] Kfoury Y et al. 2011 Tax ubiquitylation and SUMOylation control the dynamic shuttling of Tax and NEMO between Ubc9 nuclear bodies and the centrosome. Blood 117, 190–199. (doi:10.1182/blood-2010-05-285742) 20959607 10.1182/blood-2010-05-285742

[37] Martin N, Schwamborn K, Schreiber V, Werner A, Guillier C, Zhang X-D, Bischof O, Seeler J-S, Dejean A 2009 PARP-1 transcriptional activity is regulated by sumoylation upon heat shock. EMBO J. 28, 3534–3548. (doi:10.1038/emboj.2009.279) 19779455 10.1038/emboj.2009.279 PMC2782092

[38] Decque A et al. 2016 Sumoylation coordinates the repression of inflammatory and anti-viral gene-expression programs during innate sensing. Nat. Immunol. 17, 140–149. (doi:10.1038/ni.3342) 26657003 10.1038/ni.3342

[39] Garaude J, Farrás R, Bossis G, Charni S, Piechaczyk M, Hipskind RA, Villalba M 2008 SUMOylation regulates the transcriptional activity of JunB in T lymphocytes. J. Immunol. 180, 5983–5990. (doi:10.4049/jimmunol.180.9.5983) 18424718 10.4049/jimmunol.180.9.5983

[40] Zaph C et al. 2007 Epithelial-cell-intrinsic IKK-β expression regulates intestinal immune homeostasis. Nature 446, 552–556. (doi:10.1038/nature05590) 17322906 10.1038/nature05590

[41] Rimoldi M, Chieppa M, Vulcano M, Allavena P, Rescigno M 2004 Intestinal epithelial cells control dendritic cell function. Ann. NY Acad. Sci. 1029, 66–74. (doi:10.1196/annals.1309.009) 15681745 10.1196/annals.1309.009

[42] Watanabe T, Yamori M, Kita T, Chiba T, Wakatsuki Y 2005 CD4^+^ CD25^+^ T cells regulate colonic localization of CD4 T cells reactive to a microbial antigen. Inflamm. Bowel Dis. 11, 541–550. (doi:10.1097/01.MIB.0000163696.26969.e4) 15905701 10.1097/01.mib.0000163696.26969.e4

[43] Rimoldi M et al. 2005 Intestinal immune homeostasis is regulated by the crosstalk between epithelial cells and dendritic cells. Nat. Immunol. 6, 507–514. (doi:10.1038/ni1192) 15821737 10.1038/ni1192

[44] Altomare DA, Testa JR 2005 Perturbations of the AKT signaling pathway in human cancer. Oncogene 24, 7455–7464. (doi:10.1038/sj.onc.1209085) 16288292 10.1038/sj.onc.1209085

[45] Chen WS et al. 2001 Growth retardation and increased apoptosis in mice with homozygous disruption of the Akt1 gene. Genes Dev. 15, 2203–2208. (doi:10.1101/gad.913901) 11544177 10.1101/gad.913901 PMC312770

[46] Dummler B, Tschopp O, Hynx D, Yang Z-Z, Dirnhofer S, Hemmings BA 2006 Life with a single isoform of Akt: mice lacking Akt2 and Akt3 are viable but display impaired glucose homeostasis and growth deficiencies. Mol. Cell. Biol. 26, 8042–8051. (doi:10.1128/MCB.00722-06) 16923958 10.1128/MCB.00722-06 PMC1636753

[47] Ding X, Wang A, Ma X, Demarque M, Jin W, Xin H, Dejean A, Dong C 2016 Protein SUMOylation is required for regulatory T cell expansion and function. Cell Rep. 16, 1055–1066. (doi:10.1016/j.celrep.2016.06.056) 27425617 10.1016/j.celrep.2016.06.056

[48] Arranz A et al. 2012 Akt1 and Akt2 protein kinases differentially contribute to macrophage polarization. Proc. Natl Acad. Sci. USA 109, 9517–9522. (doi:10.1073/pnas.1119038109) 22647600 10.1073/pnas.1119038109 PMC3386059

[49] Scharl M, Huber N, Lang S, Fürst A, Jehle E, Rogler G 2015 Hallmarks of epithelial to mesenchymal transition are detectable in Crohn's disease associated intestinal fibrosis. Clin. Transl. Med. 4, 1 (doi:10.1186/s40169-015-0046-5) 25852817 10.1186/s40169-015-0046-5 PMC4384762

[50] Buffin-Meyer B, Crassous P-A, Delage C, Denis C, Schaak S, Paris H 2007 EGF receptor transactivation and PI3-kinase mediate stimulation of ERK by α2A-adrenoreceptor in intestinal epithelial cells: a role in wound healing. Eur. J. Pharmacol. 574, 85–93. (doi:10.1016/j.ejphar.2007.07.014) 17655843 10.1016/j.ejphar.2007.07.014

[51] Liu X et al. 2015 Knockdown of SUMO-activating enzyme subunit 2 (SAE2) suppresses cancer malignancy and enhances chemotherapy sensitivity in small cell lung cancer. J. Hematol. Oncol. 8, 67 (doi:10.1186/s13045-015-0164-y) 26063074 10.1186/s13045-015-0164-y PMC4483218

[52] Göke M, Kanai M, Lynch-Devaney K, Podolsky DK 1998 Rapid mitogen-activated protein kinase activation by transforming growth factor alpha in wounded rat intestinal epithelial cells. Gastroenterology 114, 697–705. (doi:10.1016/S0016-5085(98)70583-9) 9516390 10.1016/s0016-5085(98)70583-9

[53] Frey MR, Edelblum KL, Mullane MT, Liang D, Polk DB 2009 The ErbB4 growth factor receptor is required for colon epithelial cell survival in the presence of TNF. Gastroenterology 136, 217–226. (doi:10.1053/j.gastro.2008.09.023) 18973758 10.1053/j.gastro.2008.09.023 PMC2811086

