# Supplementary material for: SUMOylation pathway alteration coupled with downregulation of SUMO E2 enzyme at mucosal epithelium modulates inflammation in inflammatory bowel disease
Source: Open Biol. 2017 Jun 28;7(6):170024. doi: 10.1098/rsob.170024 (PMC5493774; doi:10.1098/rsob.170024)
Supplement: Supplementary material Salman et al [file rsob170024supp1.doc]

**Supplementary material**

**
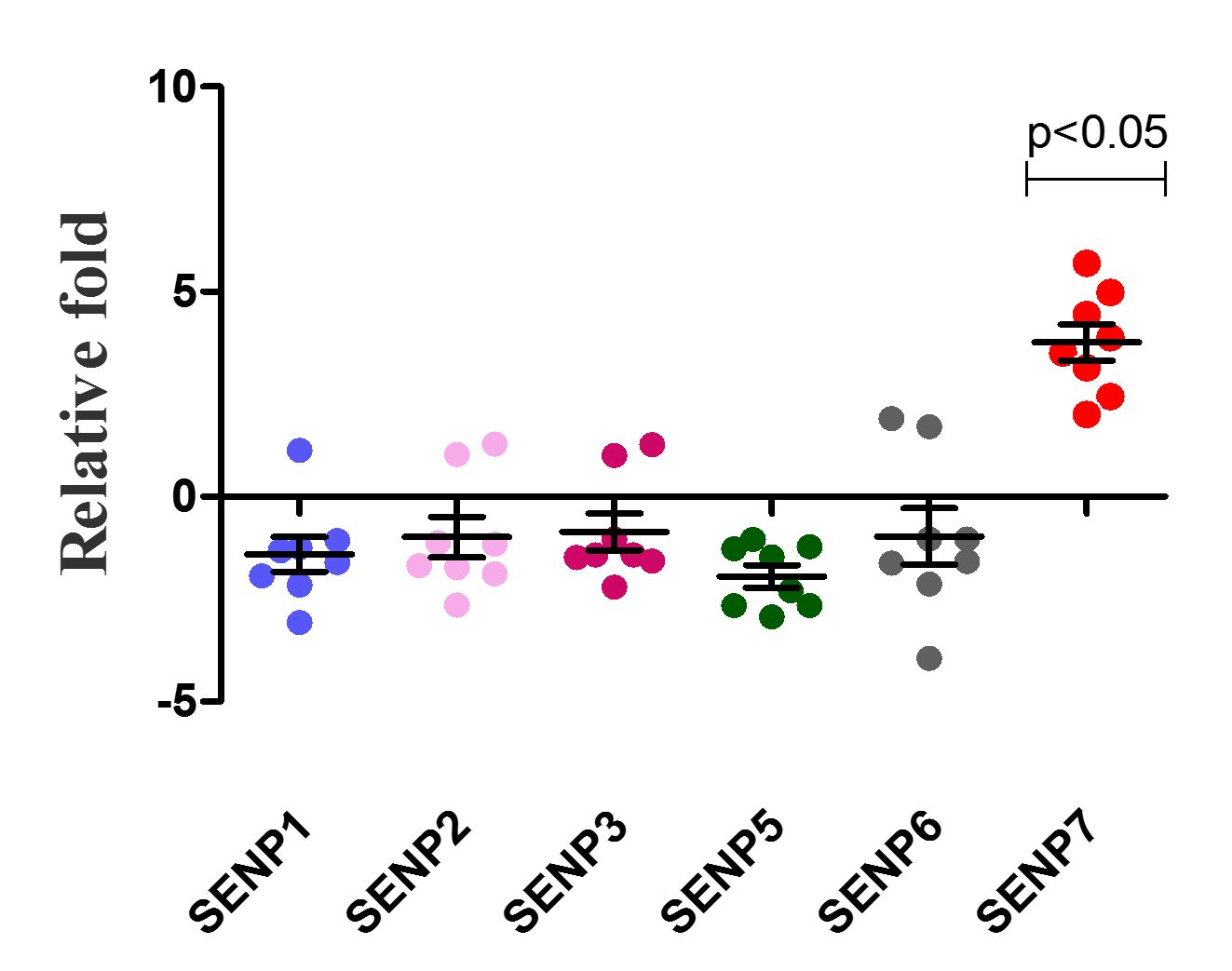
**

***FIG. S1. Expression analysis of deSUMOylases.*** *qPCR data of SENPs (SENP1, SENP2, SENP3, SENP5, SENP6, SENP7) of DSS7 mice compared to control group represented as* fold *change (*relative *to* control*), each circle represents data from one mice sample. Beta Actin and B2M were used for normalization. Data represented as Mean±SEM values of at least 3 independent experiments.*

*
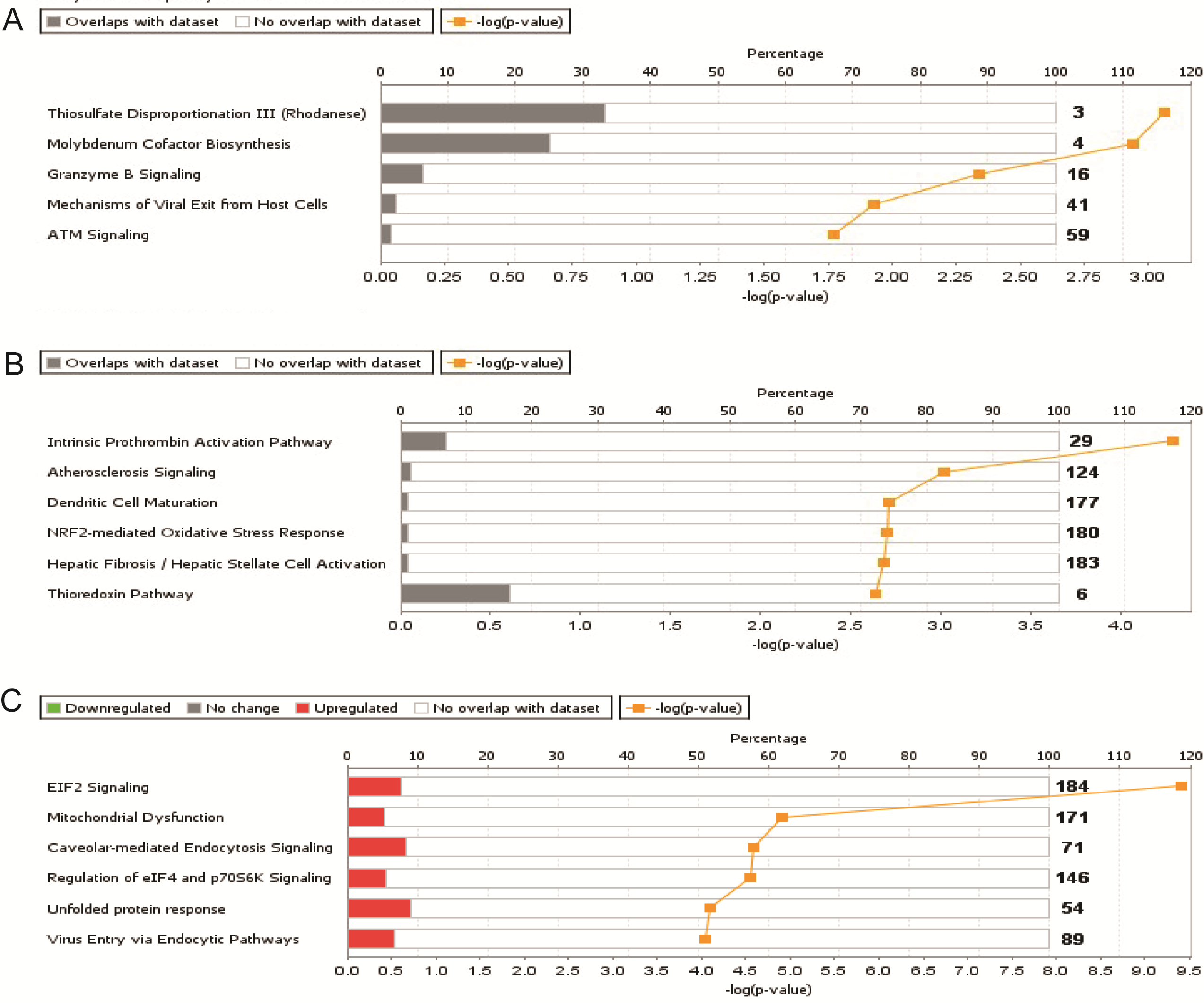
*

***FIG. S2 Ingenuity pathway analysis (IPA) of differentially regulated signalling pathways in mouse.*** *(A) And (B) Pathways associated with proteins identified exclusively in control and DSS7 mice samples. (C) Altered canonical pathways in DSS7 relative to control with fold change. Different shades of colour represent level of regulation.*

| **Protein** | **Condition of identified in current study** | **SUMOylation Position** | **Peptide** | **SIM Position** | **Peptide** | **References** |
| --- | --- | --- | --- | --- | --- | --- |
| **Akt1** | Control | 64,182,189,276 | MKTE,LKKE  AKDE,LKLE | 335-339 | LGVVM | (24) |
| **BRCA1** | Control  DSS7 Ubc9^low^  DSS7 Ubc9^hyperlow^ | 1579, 1633, 1680, 1719 | IKPE,IKTD  VKGD,PKDE | 28-32,177-181,1748-1752 | LELIK,VYIEL  LVVIV | (53) |
| **BIRC3** | Control | 23 | LKYD | - | - |  |
| **CREB1** | Control | - | - | 53-57 | VTLVQ | (54) |
| **RORy** | DSS7 Ubc9^hyperlow^ | 190  516 | AKTE  GLSK**** | 26-30  348-352 | IEVIP  IILLT | (55) |
| **NCOR1** | Control  DSS7 Ubc9^hyperlow^ | 195,261  1068,1263 | AKVE,PKVE  IKQE,IKRE | 200-204  381-385  401-405 | ILKLK  ISEII  LSVIP | (12) |
| **STAT1** | DSS7 Ubc9^hyperlow^ | 110,150, 375, 566 | LKEE,VKDQ  VKGF,IKND | - | - | (56) |
| **RXRγ** | DSS7 Ubc9^hyperlow^ | 205 | MKRE | 100-104  299-303 | LNVVN  VILLR | (57) |
| **ATG9B*** | Control  DSS7 Ubc9^low^  DSS7 Ubc9^hyperlow^ | - |  | 321-325 | VIYLH | (58) |

***Supplementary Table 1: List of proteins identified by MASCOT along with their SBM/SIM.*** *Mice samples used in the current study are indicated with the identified proteins. SBM (SUMO binding motifs) and SIM positions (SUMO interacting motifs) are shown with the peptide sequence and position. Previous SUMOylation reports of identified proteins are given with references. SUMO analysis was done using GPS-SUMO 1.0 software.*

| **Protein** | **Position/**  **SUMO binding motif** | **Position/Sumo interacting Motif** | **Disease Type** | **SUMOylation reported** |
| --- | --- | --- | --- | --- |
| BIRC3* | 23/LKYD | - | UC (regulates pro-inflammatory genes)(18) | No |
| RORy* | 190/AKTE  516/GLSK | 26-30/IEVIP  348-352/IILLT | UC and CD (regulate Th17 differentiation)(59) | No |
| STAT3 | 451/LKID  769/PKEE | 101-105/IARIV  436-440LHLIT  461-465/VVVIS | UC ( regulate anti-inflammatory genes)(60) | No |
| IL10 |  | 10-14/LVLLT | UC and CD (Anti-inflammatory)(61) | No |
| DNMT3A | 162/MKME  211/WKRE | 634-636/IRVLSV  895-899/VPVIR | CD ( methylate genes related to inflammation)(62) | Yes(63) |
| Atg16L1 | 163/LKDE | 242-246/VIVDE | UC and CD ( Autophagy related gene)(64) | No |
| NCOR1* | 195/AKVE  261/PKVE  1068/IKQE  1263/IKRE | 200-204/ILKLK  381-385/ISEII  401-405/LSVIP | UC and CD  (Anti-inflammatory molecule represses NFкB and AP-1 genes)(12) | Yes(65) |
| STAT1* | 110/ LKEE  150/ VKDQ  375/ VKGF |  | UC( pro inflammatory response)(66) | Yes(56) |
| TLR2 | 709/VKSE  783/IKSE | 733-737/ILILL | UC (pro inflammatory response)(67) | No |
| ATG9B* | - | 321-325/VIYLH | CD (autophagy gene)(58) | No |
| IL17R* | 649/AKLE | 266-270/VTLTL  380-384/VWITY  635-639/IDPLV | UC and CD (Pro inflammatory)(68) | NO |
| CARD9 | 165/LKEE  219/MAKE | 64-68/LLDIL  280-284/IQVLE | UC (regulator of NFκB and apoptosis)(69) | NO |
| IL23R |  | 147-151/LSLIL | UC and CD (pro inflammatory)(69) | NO |
| IRGM |  | 86-90/VVLWD | CD (Autophagy pathway gene)(64) | No |
| PTPN2 |  | 73-77/LVDIE  233-237/LVLME  397-401/VILVG | CD (Regulation of epithelial barrier function)(70) | No |
| PPARγ | 79/IKVE  367/PKFE | 315-319/VTLLK  388-392/IAVII | UC and CD ( express in IEC and maintain immune response in mucus)(71) | Yes(12) |

˄Protein identified in our study

***Supplementary Table 2: List of proteins having connection with IBD and SUMOylation.*** *Table shows the list of important proteins having direct role in UC and CD disease along with their SUMOylation reports. SBM (SUMO binding motifs) and SIM positions (SUMO interacting motifs) along with the peptide sequence and position are represented.*

**Primers Sequences:**

Mice primers

| B2M F | TTCTGGTGCTTGTCTCACTGA |
| --- | --- |
| B2M R | CAGTATGTTCGGCTTCCCATTC |
| IL8 F | CACCTCAAGAACATCCAGAGCT |
| IL8 R | CAAGCAGAACTGAACTACCATCG |
| B-Actin F | TCTACGAGGGCTATGCTCTCC |
| B-Actin R | GGATGCCACAGGATTCCATAC |
| UBC9 F | GTACCTGGAAGAGGCAGCTC |
| UBC9 R | TAGGGCAGAGTTGAATGCAG |

Human Primers

| HPRT F | GCTATAAATTCTTTGCTGACCTGCTG |
| --- | --- |
| HPRT R | AATTAACTTTTATGTCCCCTGTTGACTGG |
| IL8 F | GGCACAAACTTTCAGAGACAGCAG |
| IL8 R | GTTTCTTCCTGGCTCTTGTCCTAG |
| GAPDH F | CTCACCGGATGCACCAATGTT |
| GAPDH R | CGCGTTGCTCACAATGTTCATY1 |
| UBC9 F | ATGGAGGAAAGACCACCCATTTG |
| UBC9 R | CCACGGAGTCCCTTTCTTTCC |
| c-FOS F | AACCTGTCAAGAGCATCAGC |
| c-FOS R | ATGATGCTGGGAACAGGAAG |
| c-JUN F | TCCACGGCCAACATGCT |
| c-JUN R | CCACTGTTAACGTGGTTCATGAC |
| BIRC3 F | AGAATTGGCAAGAGCTGGTT |
| BIRC3 R | TACTGAGCTTCCCACCACAG |
| ICAM F | GTGCTGGTGAGGAGAGATCA |
| ICAM R | GGTGTTCTCAAACAGCTCCA |
| AKT1 F | GGTCTGTAACCACCCTGGAC |
| AKT1 R | CGCTGGAGACAAAGAGAGGT |
| PPARγ F | CGAGGACACCGGAGAGGG |
| PPARγ R | TGTGGTTTAGTGTTGGCTTCTT |
| RelA/p65 F | GGAAAAGACTGCAGAGACGG |
| RelA/p65 R | ATTGGGTTCGAGACAACAGG |
| TNF α F | AATAGGCTGTTCCCATGTAGC |
| TNF α R | AGAGGCTCAGCAATGAGTGA |
| SAE2 F | TAAGGATGACCCATCTGCAA |
| SAE2 R | TCCCTGCCATTGATTTGATA |
| Cx43 F | GGATCGGGTTAAGGGAAAGAG |
| Cx43 R | AGGAGACATAGGCGAGAGG |
| NOS2 F | GTTTGACCAGAGGACCCAG |
| NOS 2 R | ATCTCCTTTGTTACCGCTTCC |
| TNFAIP3 F | TGTTAATGCCTCTGAGTGTCC |
| TNFAIP3 R | TCTTTCCCTGTGACCATTGC |
| TNFAIP8 F | CTATCAGTGGCAGCTCTTGTC |
| TNFAIP8 R | CCCCTACAAATTGGCTGAGAG |
| PTGES F | CAAGTGTATGGTGGGAAGGAG |
| PTGES R | TCGCGGACAATGTAGTCAAAG |

**Plasmid List:**

1) Ubc9 expressing plasmid – pCDNA3/Ubc9 (Plasmid # 20082, addgene)

2) WT-Akt-1 – pCDNA3-flag-HA-Akt-1 (Plasmid # 9021, addgene)

3) DN-Akt-1 - HA-Akt DN (K179M) (Plasmid # 16243, addgene)

4) SMUT-Akt1 - pCDNA3-flag-HA-Akt-1 (K276). Mutation was confirmed by sequencing.
